# Long-term in vivo application of a potassium channel-based optogenetic silencer in the healthy and epileptic mouse hippocampus

**DOI:** 10.1186/s12915-021-01210-1

**Published:** 2022-01-14

**Authors:** P. Kleis, E. Paschen, U. Häussler, Y. A. Bernal Sierra, C. A. Haas

**Affiliations:** 1 Experimental Epilepsy Research, Department of Neurosurgery, Medical Center - University of Freiburg, Faculty of Medicine, 79106 Freiburg, Germany; 2 Faculty of Biology, University of Freiburg, 79104 Freiburg, Germany; 3 BrainLinks-BrainTools, University of Freiburg, 79110 Freiburg, Germany; 4 Experimental Biophysics, Institute of Biology, Humboldt University of Berlin, 10115 Berlin, Germany; 5 Center for Basics in NeuroModulation, Faculty of Medicine, University of Freiburg, 79106 Freiburg, Germany

**Keywords:** bPAC, cAMP, Electrophysiology, Epilepsy, Kainate, Optogenetic inhibition

## Abstract

**Background:**

Optogenetic tools allow precise manipulation of neuronal activity via genetically encoded light-sensitive proteins. Currently available optogenetic inhibitors are not suitable for prolonged use due to short-lasting photocurrents, tissue heating, and unintended changes in ion distributions, which may interfere with normal neuron physiology. To overcome these limitations, a novel potassium channel-based optogenetic silencer, named PACK, was recently developed. The PACK tool has two components: a photoactivated adenylyl cyclase from *Beggiatoa* (bPAC) and a cAMP-dependent potassium channel, SthK, which carries a large, long-lasting potassium current in mammalian cells. Previously, it has been shown that activating the PACK silencer with short light pulses led to a significant reduction of neuronal firing in various in vitro and acute in vivo settings. Here, we examined the viability of performing long-term studies in vivo by looking at the inhibitory action and side effects of PACK and its components in healthy and epileptic adult male mice.

**Results:**

We targeted hippocampal *cornu ammonis* (CA1) pyramidal cells using a viral vector and enabled illumination of these neurons via an implanted optic fiber. Local field potential (LFP) recordings from CA1 of freely moving mice revealed significantly reduced neuronal activity during 50-min intermittent (0.1 Hz) illumination, especially in the gamma frequency range. Adversely, PACK expression in healthy mice induced chronic astrogliosis, dispersion of pyramidal cells, and generalized seizures. These side effects were independent of the light application and were also present in mice expressing bPAC without the potassium channel. Light activation of bPAC alone increased neuronal activity, presumably via enhanced cAMP signaling. Furthermore, we applied bPAC and PACK in the contralateral hippocampus of chronically epileptic mice following a unilateral injection of intrahippocampal kainate. Unexpectedly, the expression of bPAC in the contralateral CA1 area was sufficient to prevent the spread of spontaneous epileptiform activity from the seizure focus to the contralateral hippocampus.

**Conclusion:**

Our study highlights the PACK tool as a potent optogenetic inhibitor in vivo. However, further refinement of its light-sensitive domain is required to avoid unexpected physiological changes.

**Supplementary Information:**

The online version contains supplementary material available at 10.1186/s12915-021-01210-1.

## Background

Cell type-specific inhibition techniques are required in neuroscience to investigate the contribution of neuronal populations in physiological and pathophysiological processes. Optogenetic silencing takes advantage of genetically encoded light-sensitive proteins, allowing to “switch off” neurons of interest with high temporal and spatial precision. Currently available optogenetic inhibitors such as inward-directed chloride pumps (e.g., halorhodopsins) and outward-directed proton pumps (e.g., archaerhodopsins) have several limitations. Namely, they require continuous high-intensity illumination, which can have unexpected excitatory outcomes due to tissue heating, abnormal ion distributions, and strong rebound responses [1, 2]. Even brief activation of halorhodopsin can change the intracellular chloride concentration, cause a positive shift in γ-aminobutyric acid A (GABA_A_) receptor reversal potential, and decrease the action potential threshold, leading to elevated network excitability [3–5].

Using potassium (K^+^) current as the hyperpolarizing factor would be a better approach since the resting state of neurons is based on K^+^ conductance and is thus more physiological than pumping chloride or protons against their electrochemical gradients. Several synthetic light-activated K^+^ channels have been engineered which, however, pose shortcomings such as the requirement of a chemical cofactor [6, 7], utilizing UV light [8], or very low photocurrents in mammalian cells [9]. A newly developed K^+^ channel-based optogenetic silencer could potentially overcome these limitations [10, 11]. The two-component silencer, named PACK, comprises a soluble photoactivated adenylyl cyclase from the *Beggiatoa* bacterium (bPAC [12]) and a cyclic nucleotide-gated potassium channel from *Spirochaeta thermophila* (SthK [13];). The blue light receptor in bPAC activates the cyclase domain thus increasing cytosolic cyclic adenosine monophosphate (cAMP), which subsequently opens the co-expressed SthK channels. Benefits of the PACK silencer include robust expression in mammalian cells, signal amplification, and large long-lasting K^+^ currents. Previously, PACK has been shown to reliably inhibit neuronal firing in cell cultures, acute slice preparations, and anesthetized mice [11]. However, a long-term application in awake mice has so far not been tested. A prolonged precise inhibition technique would be valuable for investigating the contribution of specific cell populations in pathologies such as epilepsy.

Mesial temporal lobe epilepsy (MTLE), the most common type of acquired focal epilepsy in adults, is characterized by spontaneous hippocampal seizures, which are often pharmacoresistant [14, 15]. MTLE is usually described as a unilateral disease since the seizures arise in one hemisphere, ipsilateral to the pathological abnormality. However, in some patients and MTLE mouse models, epileptic activity propagates to the contralateral hippocampus [16–20]. To scrutinize the performance of PACK and investigate the contribution of the contralateral hippocampus in MTLE, we applied PACK-mediated inhibition in the well-established intrahippocampal kainate (ihpKA) mouse model. This model recapitulates the major pathological features of human MTLE, such as unilateral hippocampal sclerosis with cell loss and gliosis accompanied by subclinical spontaneous seizures [21–23]. Contralateral CA1 cells in the ihpKA mouse model exhibit elevated activity-related cytoskeleton (Arc) gene expression during the chronic phase of epilepsy [24], suggesting that these cells are involved in the contralateral epileptiform activity.

Here, we aimed to validate the inhibitory action of the PACK silencer in principal neurons of hippocampal CA1 in freely moving mice. We investigated the long-term histological and electrophysiological effects of the PACK construct and its components, the viral vector with a fluorescent marker (mCherry) and the adenylyl cyclase bPAC, in vivo. We present evidence that PACK activation persistently reduces neuronal activity during illumination with a frequency as low as 0.1 Hz. Furthermore, we applied the PACK silencer in chronically epileptic mice, where PACK expression in CA1, contralateral to the seizure focus, prevented seizure spread between hemispheres.

## Results

### Light-activated PACK reduces the activity of pyramidal cells in vivo

**Fig. 1:**
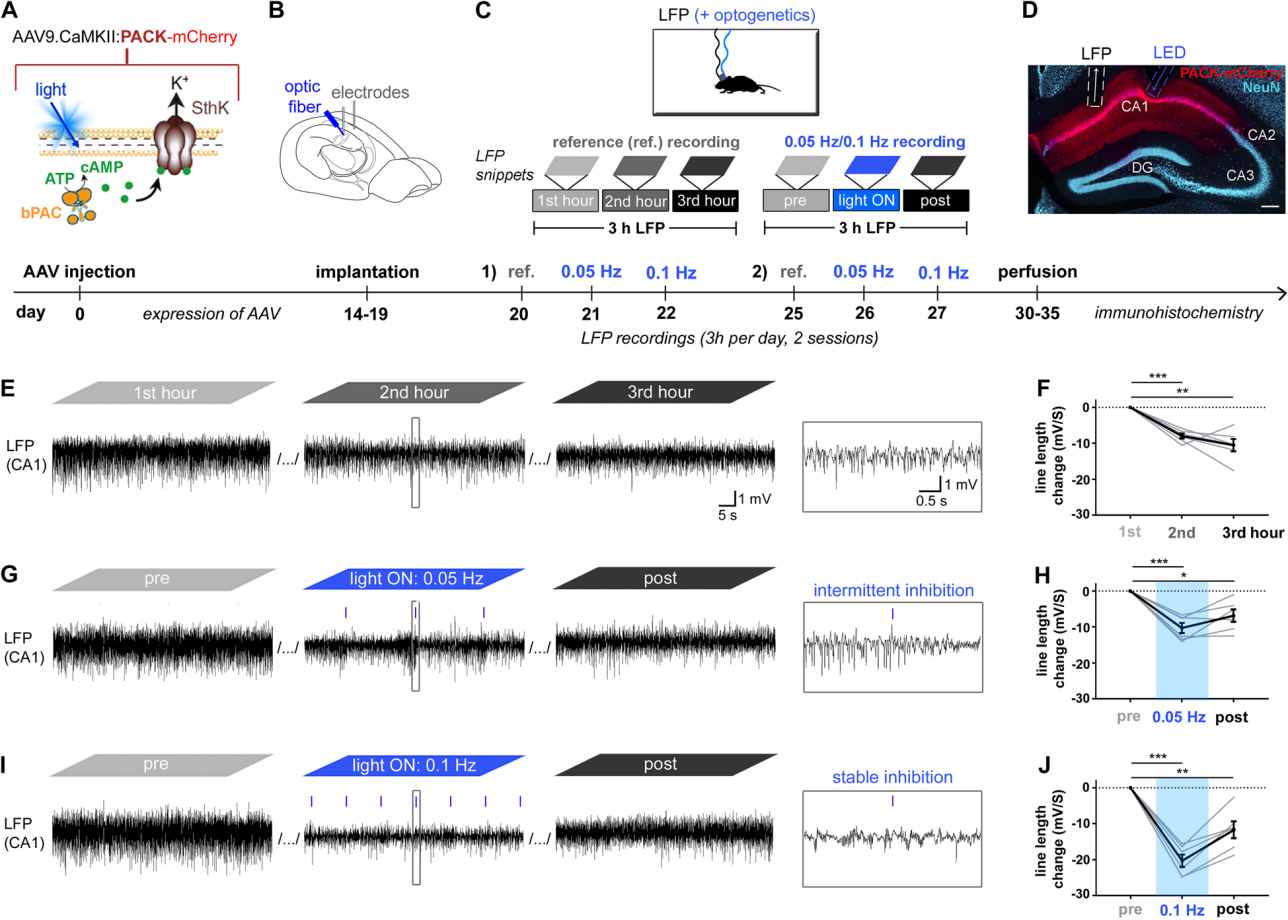
PACK-mediated optogenetic inhibition of CA1 principal cells in vivo. **A**–**D** Experimental design with a timeline. **A** We targeted PACK (bPAC+Sthk) to excitatory neurons by injecting the AAV9.CaMKIIα vector into the CA1 region of the hippocampus. **B** After implantations of wire electrodes and an optic fiber into CA1, **C** we recorded LFPs in freely moving mice for 3 h a day. Reference (ref.) recordings were without illumination, whereas the “0.05 Hz/0.1 Hz recordings” included 1-h pre-recording, light ON phase with 0.05-Hz or 0.1-Hz illumination, and a post-recording. Each recording type was repeated twice and the mean of the two sessions is presented for each animal. **D** PACK-mCherry expression and electrode/optic fiber positions in CA1 were confirmed by histology at the end of the experiment. **E**, **G**, **I** Representative LFP snippets from the first, second, and third hour of each recording type. **E** In ref. recordings, LFP magnitude decreased over time, **F** confirmed by the significant drop of mean line length (in black) in the second and third recording hour. **G** Applying 10-ms blue light pulses (~ 80 mW/mm^2^, 460 nm) at 0.05 Hz resulted in an intermittent reduction of LFP magnitude. **H** Mean line length was significantly reduced during 0.05-Hz light application and in the post-recording. **I** Applying light pulses at 0.1 Hz resulted in a stable reduction of LFP magnitude, **J** which is reflected in a robust reduction of line length during 0.1-Hz illumination. One-sample *t*-test (*n* = 6 mice, average of 2 recordings, gray lines), *p < 0.05, **p < 0.01, ***p < 0.001. Mean presented in black with SEM as error bars

To verify the inhibitory action of the PACK silencer in awake mice, we targeted hippocampal principal cells by locally injecting AAV9.CaMKIIα.PACK-mCherry into the CA1 area of the dorsal hippocampus (Fig. 1A) and enabled illumination onto these neurons via an implanted optic fiber (Fig. 1B). To test whether applying short light pulses (10 ms) at low frequencies in vivo results in sustained inhibition of PACK-expressing CA1 neurons as previously demonstrated in vitro [11], we shined blue light at 0.05 Hz and 0.1 Hz for 1 h. The light ON phase was enclosed by a pre- and post-recording, 1 h each (Fig. 1C). Following the recording phase, histological analysis revealed that the expression of PACK-mCherry was restricted to pyramidal neurons in CA1 with labeling in cell bodies and dendrites (Fig. 1D). For LFP analysis, we only included mice, which had the optic fiber and recording electrode positioned in CA1 (Additional file 1: Fig. S1, *n* = 6).

First, we recorded 3-h reference LFPs in each mouse (Fig. 1E) to control for the change in LFP characteristics occurring without blue light application. Quantification of the LFP signal by determination of the line length, a measure for LFP magnitude (see the “Methods” section), revealed a decrease during the reference recordings in PACK mice (first hour set to zero; second hour −7.98 ± 1.71 mV/s, one-sample *t*-test: *t* = 11.40, *n* = 6, *p* < 0.0001, *α* = 0.025; third hour −10.54 ± 4.21 mV/s, one-sample *t*-test: *t* = 6.313, *n* = 6, *p* = 0.0017, *α* = 0.025; Fig. 1F). Next, we activated PACK with intermittent light application. During 0.05-Hz illumination, LFP magnitude was reduced directly after light pulses, followed by periods of recovery (Fig. 1G). The mean line length significantly decreased during 0.05-Hz illumination (50 min) compared to the pre-recording (−10.33 ± 1.41 mV/s, one-sample *t*-test: *t* = 7.34, *n* = 6, *p* = 0.0007, *α* = 0.025; Fig. 1H). Illumination with 0.1 Hz provided a stable reduction of the LFP magnitude (Fig. 1I) with a strong decrease in the line length during the light ON phase (−20.30 ± 1.73 mV/s, one-sample *t*-test: *t* = 11.75, *n* = 6, *p* < 0.0001, *α* = 0.025; Fig. 1J). Since there was a drop of line length already in recordings without light application, we compared the change of line length from the first to the second hour in the reference recordings to the sessions with 0.05-Hz and 0.1-Hz illumination, revealing a significant difference (reference −7.98 ± 0.70 mV/s, 0.05 Hz −10.33 ± 1.41 mV/s, 0.1 Hz −20.30 ± 1.73 mV/s, RM ANOVA: *F* = 24.35, *n* = 6, *p* = 0.0019). The reduction of line length was notably higher in the 0.1-Hz recordings than in the respective reference recordings (Tukey’s multiple comparison test: *p* = 0.003), whereas in the sessions with 0.05 Hz, it was similar to the reference recordings (Tukey’s multiple comparison test: *p* = 0.09).

**Fig. 2:**
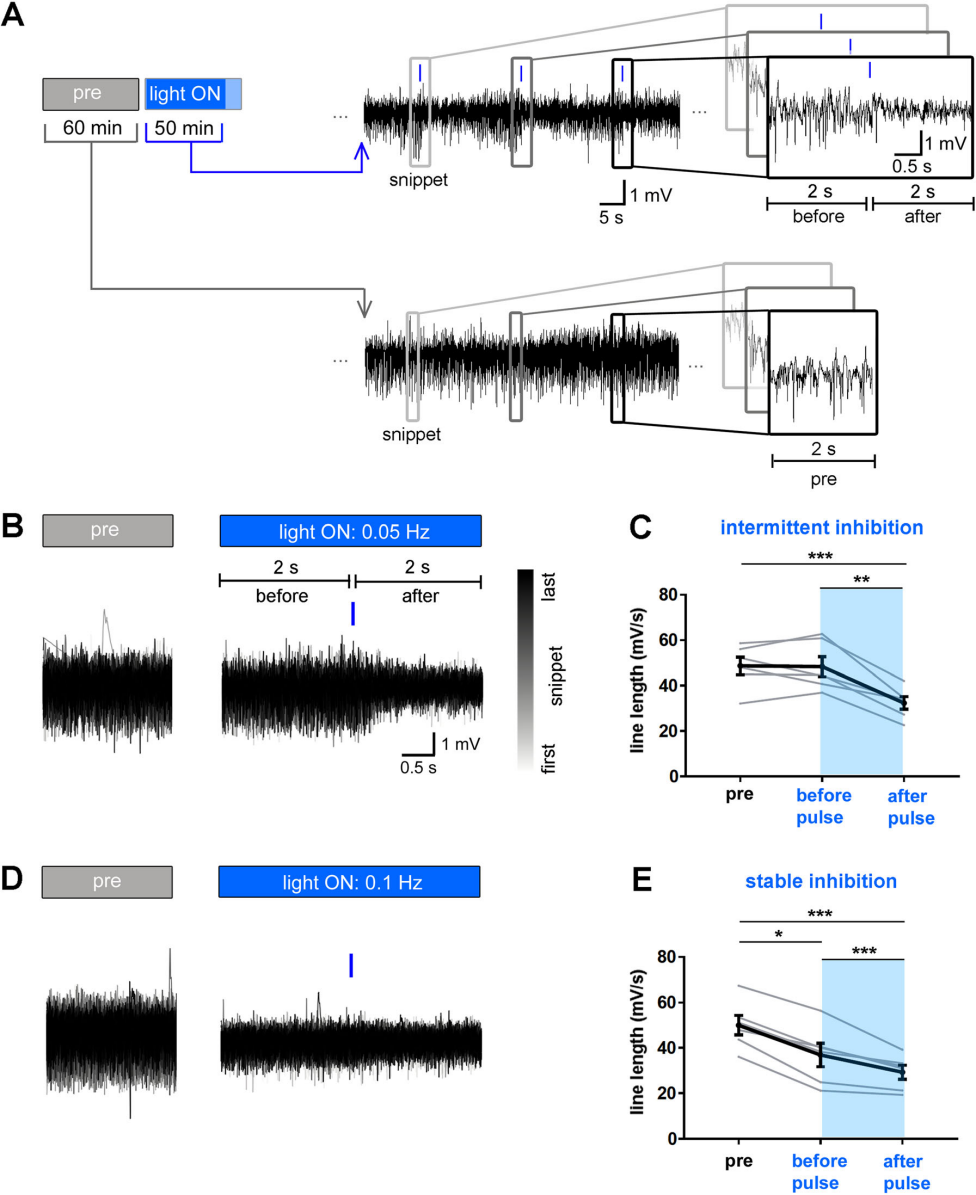
PACK-mediated inhibition in vivo is reliable and reversible. **A** Analysis of responses to 10-ms blue light pulses during the first 50 min of the light ON phase. 2-s long LFP snippets before and after each light pulse were extracted and overlaid with a color-coding from gray to black (first to last LFP snippet). To enable comparison to baseline, 2-s snippets at corresponding time points were extracted from pre-recordings. **B**, **D** Representative plots of overlaid LFP snippets from a pre-recording and light ON recording with **B** 0.05-Hz and **D** 0.1-Hz illumination. **C**, **E** Line lengths (mV/s) were calculated for each 2-s LFP snippet and the mean “pre,” “before pulse,” and “after pulse” line length per mouse is presented. The mean “after pulse” line length was significantly smaller than “pre” and “before pulse” line length in both illumination modes, indicating a reliable reduction of neuronal activity. **C** Baseline (“pre”) and “before pulse” line lengths were similar in 0.05-Hz sessions, demonstrating recovery from inhibition before the next pulse was applied. **E** LFP line length was reduced throughout the 0.1-Hz light ON period (in blue), including before and after pulse phases, suggesting the inhibition was stable. RM ANOVA and Tukey’s multiple comparison test (*n* = 6 mice, average of 2 recordings, gray lines), *p < 0.05, **p < 0.01, ***p < 0.001. Mean presented in black with SEM as error bars

To test the reliability of the PACK-mediated inhibition, we further analyzed the responses to light pulses applied at 0.05 Hz and 0.1 Hz. We extracted 2-s LFP snippets before and after each light pulse (Fig. 2A), plotted their overlay (Fig. 2B, D), and calculated the mean of the “before pulse” and “after pulse” line lengths for each recording session (Fig. 2C, E). We also extracted 2-s LFP snippets at corresponding time points from respective pre-recordings (“pre”) to serve as a baseline. In the 0.05-Hz session, reduction of LFP magnitude was reliable and reversible since there was a reduction after each light pulse, followed by a complete recovery during the 20-s interval between subsequent pulses (Fig. 2B). The mean “after pulse” line length was significantly smaller than “pre” and “before pulse” line length in 0.05-Hz sessions (“pre” 48.67 ± 3.88 mV/s, “before pulse” 48.38 ± 4.40 mV/s, “after pulse” 32.39 ± 2.74 mV/s, RM ANOVA: *F* = 28.65, *n* = 6, *p* = 0.0005, Tukey’s multiple comparison test: *p* < 0.01; Fig. 2C). Shining 10-ms light pulses every 10 s provided a persistent reduction in LFP amplitude (Fig. 2D). In the 0.1-Hz sessions, the line length was lower during the whole light ON period, including “after pulse” and “before pulse” snippets, suggesting a constant inhibitory effect (“pre” 49.97 ± 4.30 mV/s, “before pulse” 36.89 ± 5.16 mV/s, “after pulse” 29.31 ± 3.10 mV/s, RM ANOVA: *F* = 61.23, *n* = 6, *p* < 0.0001, Tukey’s multiple comparison test: “pre” vs. “before pulse” *p* = 0.0009, “pre” vs. “after pulse” *p* = 0.002, “before pulse” vs. “after pulse” *p* = 0.04; Fig. 2E). In summary, applying blue light at 0.1 Hz onto PACK-expressing CA1 neurons in vivo resulted in a sustained reduction of the net neuronal activity.

**Fig. 3:**
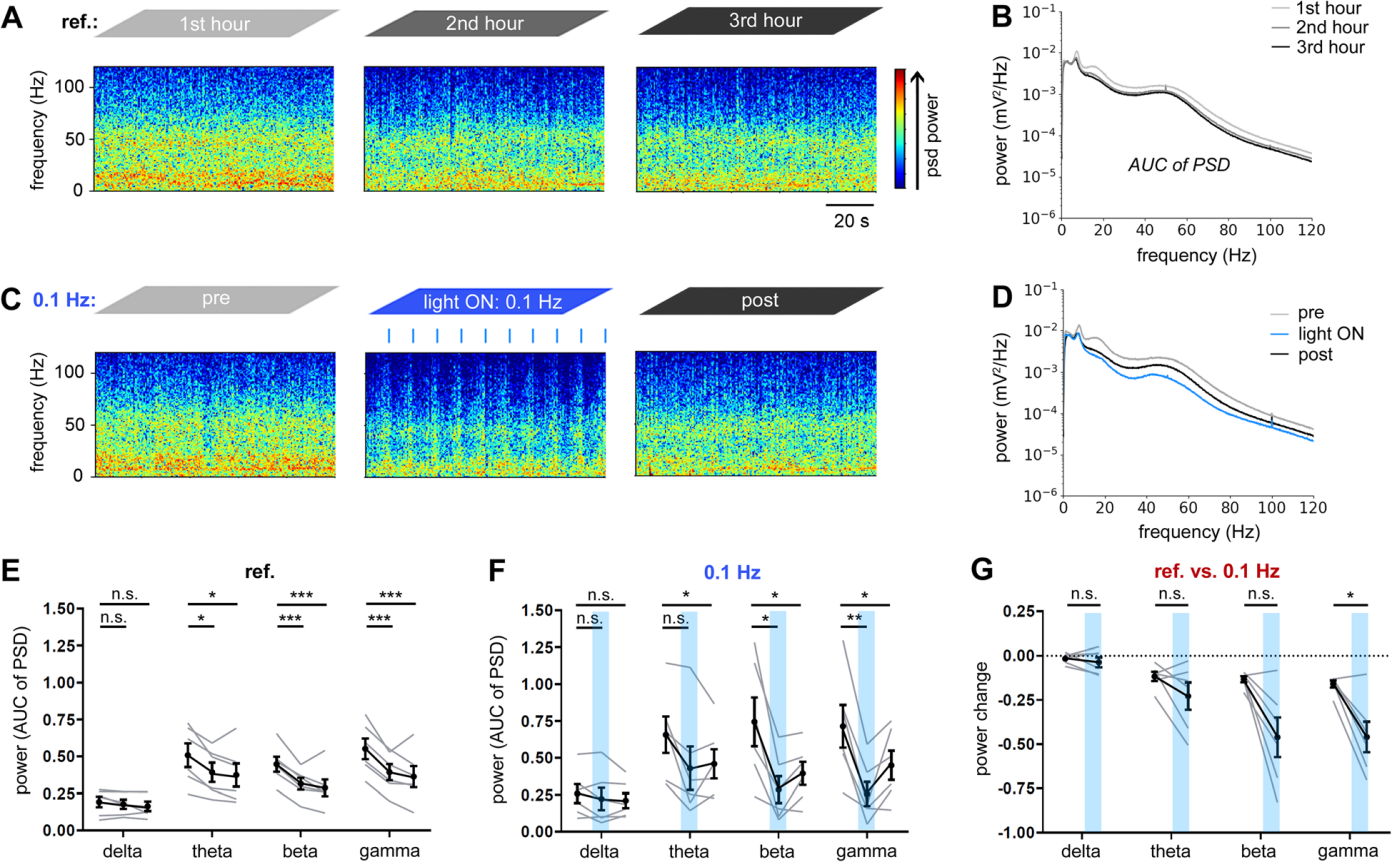
Spectral analysis reveals reduced power of gamma oscillations during light activation of PACK. **A** Representative spectrogram snippets from a ref. recording show baseline activity; blue colors reflect low power and red colors reflect high power of the corresponding frequencies. **B** Mean power spectral density (PSD) during the first (light gray), second (dark gray), and third (black) recording hour. **C** Light pulses applied at 0.1 Hz transiently decreased the spectral power. **D** The mean PSDs pre- (light gray), light ON (blue), and post-recordings (black). **E**–**G** The oscillatory power was calculated by taking the AUC of the PSD plot in each frequency range: delta (1–4 Hz), theta (4–12 Hz), beta (12–30 Hz), and gamma (30–120 Hz). During the **E**ref. recordings, the power of theta, beta, and gamma oscillations declined significantly. **F** During 0.1-Hz illumination, the power of beta and gamma oscillations also decreased notably. Two-way RM ANOVA, Dunnett’s multiple comparison test (*n* = 6 mice, average of two recordings, gray lines). **G** Power change from the first to the second hour in ref. versus 0.1-Hz recordings revealed that only gamma power was further reduced by the light-induced PACK activation. Multiple paired *t*-tests with an adjusted significance level (*α* = 0.0125). Mean presented in black with SEM as error bars

Next, we examined how light-induced PACK activation in principal cells alters oscillatory activity in CA1. The most dominant oscillations measured in the hippocampus of freely behaving rodents are theta (4–10 Hz), beta (12–30 Hz), and gamma (30–120 Hz) waves [25–27]. The theta and gamma peaks are prominent in the spectrogram snippets (Fig. 3A) and in the mean power spectral density (PSD) plot (PSD averaged across recordings; Fig. 3B) of the reference recordings acquired from PACK mice. We quantified the oscillatory power by taking the area under the curve (AUC) of the PSD plot in the respective frequency range. Similarly to the line length, the power of theta, beta, and gamma oscillations decreased significantly within the 3-h reference recordings (two-way RM ANOVA, Dunnett’s multiple comparison test, *n* = 6, Additional file 2: Table S1; Fig. 3E).

Applying light pulses at 0.1 Hz transiently altered the spectral power (Fig. 3C). The mean PSD during the 50-min 0.1-Hz light ON phase was visibly reduced compared to pre- and post-recordings, especially in frequencies above ~ 10 Hz (Fig. 3D). Quantification of the power change revealed a significant drop of beta and gamma power during 0.1-Hz illumination (two-way RM ANOVA, Dunnett’s multiple comparison test, *n* = 6, Additional file 2: Table S1, Fig. 3F). However, only the reduction of gamma power was significantly stronger during 0.1-Hz illumination than the decline in respective reference recordings (multiple paired *t*-test, gamma: *t* = 3.94, *n* = 6, *p* = 0.01, *α* = 0.0125; Fig. 3G). These data indicate that PACK-mediated inhibition of CA1 neurons in vivo alters mainly the power of gamma oscillations.

### Light-dependent hyperactivity in bPAC-expressing mice

Activation of the PACK silencer includes cAMP production by soluble bPAC, which then opens the co-expressed SthK potassium channels in the cell membrane. The second messenger molecule, cAMP, is an important component of intracellular signaling, regulating the plasticity and excitability of neurons [28–31]. Therefore, it is crucial to investigate whether activation of bPAC alone affects network excitability.

**Fig. 4:**
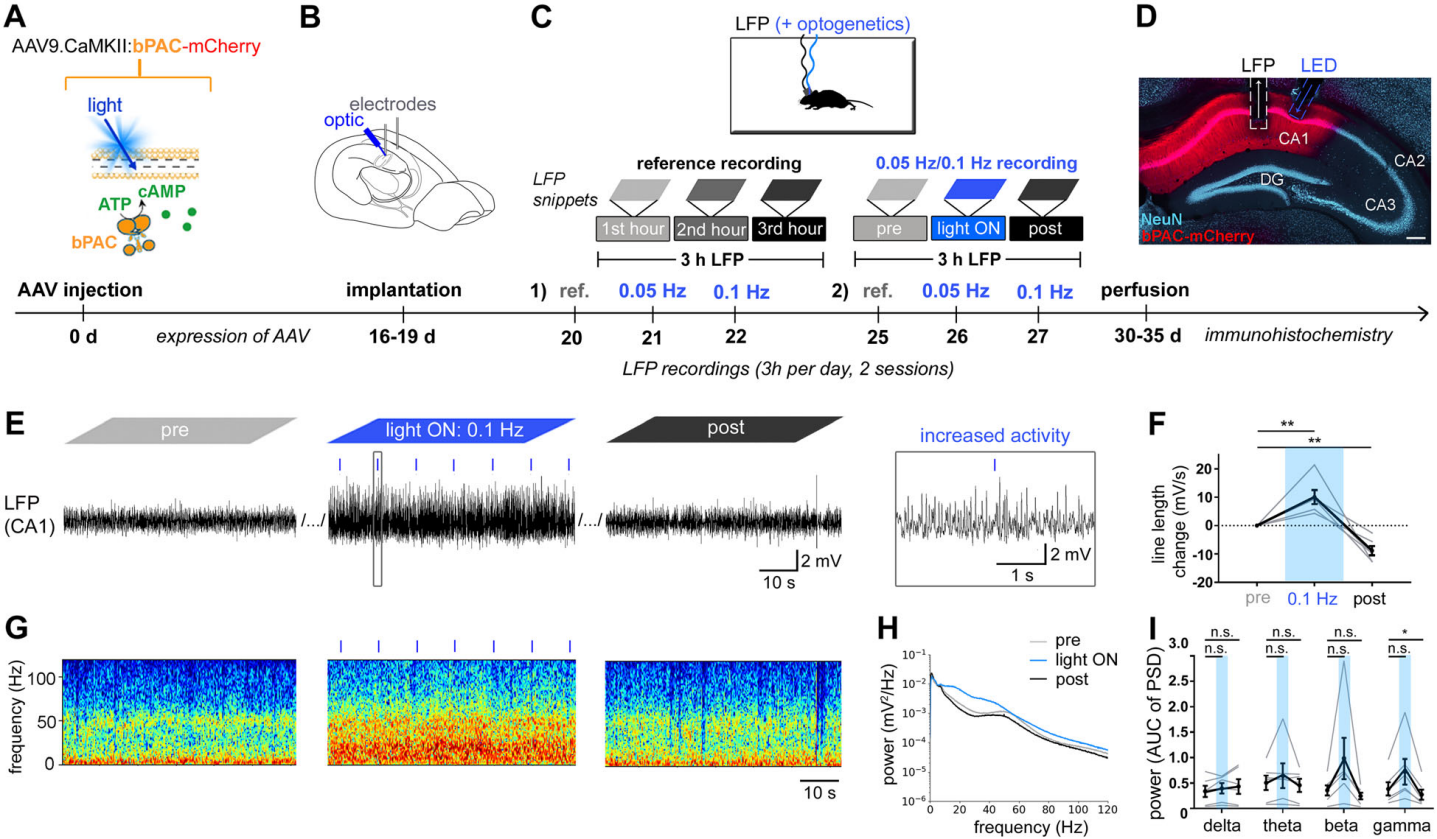
Light activation of bPAC reversibly increases neuronal activity in CA1. **A** Experimental design. We targeted bPAC-mCherry to excitatory neurons in the CA1 area using the AAV9 vector. **B** Implantation, **C** recordings, and **D** histological analysis were performed like in PACK mice. Scale bar: 200 μm. **E** Representative snippets of a 0.1-Hz recording show persistently increased LFP magnitude during the light ON phase in bPAC-expressing CA1. **F** In the light ON phase, the mean line length was significantly increased compared to the baseline (pre) level. In the post-recordings, the line length was reduced compared to the baseline. One-sample *t*-test (*n* = 6, average of two recordings, gray lines), **p < 0.01. **G** The spectrograms, taken from the same time window as LFP snippets, demonstrate elevated spectral power. **H** The mean PSD during 0.1-Hz illumination was increased at frequencies above ~ 10 Hz. **I** Theta (4–12 Hz), beta (12–30 Hz), and gamma (30–120 Hz) powers are slightly but not significantly elevated during the light ON phase. Two-way RM ANOVA, Dunnett’s multiple comparison test, **p < 0.05. Mean presented in black with SEM as error bars

To this end, we targeted bPAC with the AAV9.CaMKIIα viral vector to CA1 neurons and repeated the experiments like with PACK mice (Fig. 4A–D). During the reference recordings, the mean line length dropped similarly as in PACK mice (first hour set to zero, second hour −8.45 ± 0.90 mV/s, one-sample *t*-test: *t* = 9.43, *n* = 6, *p* < 0.0001, *α* = 0.025; third hour, −10.40 ± 4.49 mV/s, one-sample *t*-test: *t* = 8.02, *n* = 6, *p* < 0.0001, *α* = 0.025; Additional file 1: Fig. S3A). The changes in spectral activity included a reduction in gamma power, whereas other oscillations were not consistently altered during the reference recordings (Additional file 2: Table S2, Additional file 1: Fig. S3B-C). In the reference recordings from control mice injected with AAV9.CaMKIIα.mCherry (mCherry mice), a slight decrease in line length and a significant reduction of gamma power were also evident (Additional file 1: Fig. S2E-I). Therefore, the run-down of LFP signal in reference recordings can be attributed to habituation-reduction in arousal and exploratory behavior [32, 33].

Surprisingly, light activation of bPAC at 0.1 Hz led to sustained neuronal hyperactivity that was clearly visible in LFP snippets (Fig. 4E) as well as in corresponding spectrograms (Fig. 4G). The line length significantly increased during 0.1-Hz illumination (10.08 ± 2.46, one-sample *t*-test: *t* = 4.09, *n* = 6, *p* = 0.009, *α* = 0.025; Fig. 4F). In the post-recording, LFP magnitude dropped again, suggesting that the neuronal hyperactivity was reversible and related to light-induced elevation of cAMP levels (line length −8.86 ± 2.80 mV/s, one-way *t*-test: *t* = 3.16, *n* = 12, *p* = 0.0091, *α* = 0.025; Fig. 4F). Mainly beta and gamma oscillations were amplified by bPAC activation, although due to high variability, there were no significant differences between the pre-recording and the light ON phase (Additional file 2: Table S2, Fig. 4H, I). The elevation in neuronal activity was not induced by blue light *per se *since mice injected with AAV9.CaMKIIα.mCherry did not show any changes in the LFP signal during illumination (Additional file 1: Fig. S2J-N). In summary, light-induced activation of bPAC transiently elevates neuronal activity in the hippocampal CA1.

### Spontaneous generalized seizures arising in PACK- and bPAC-expressing mice

**Fig. 5:**
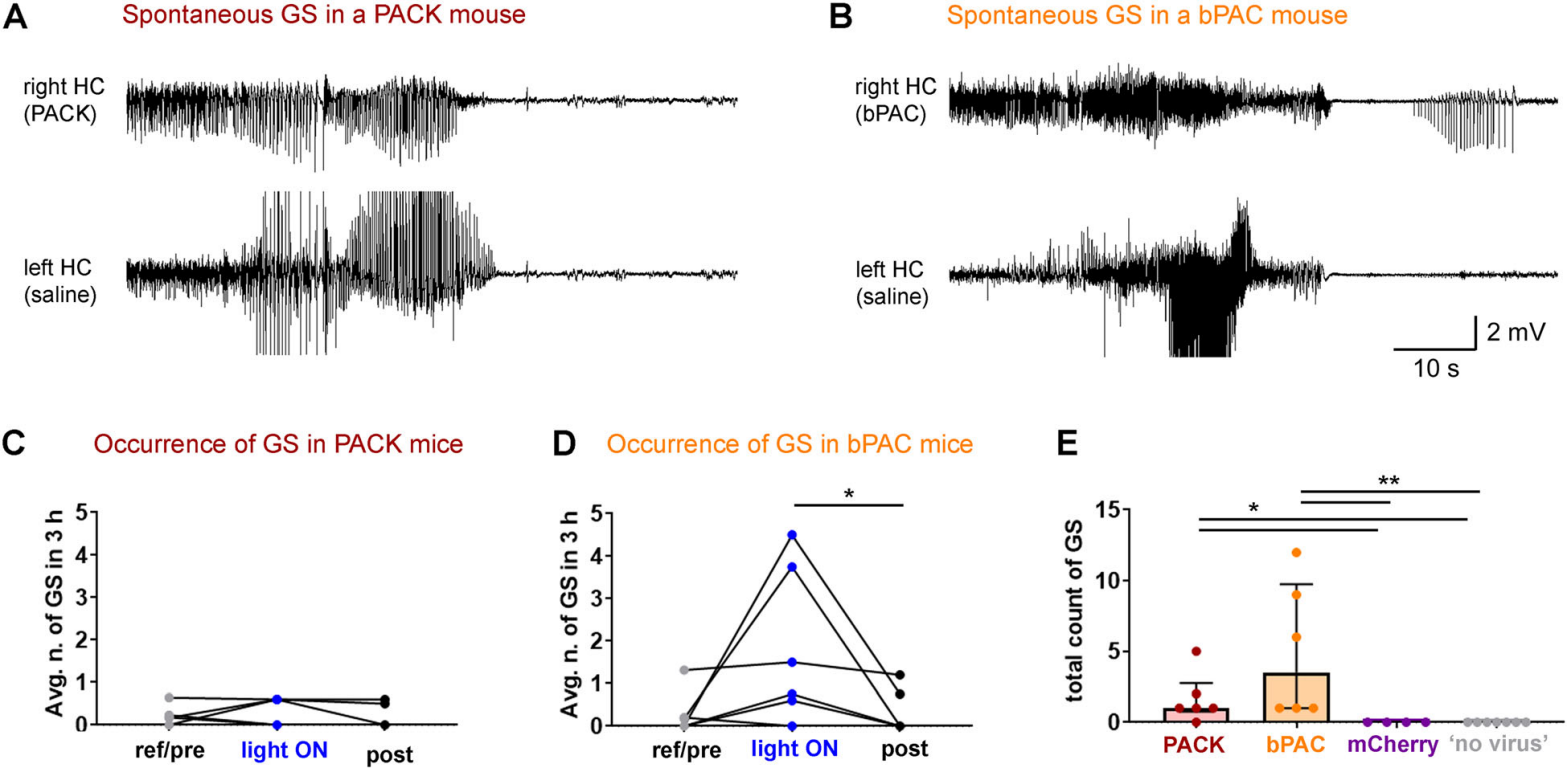
Spontaneous generalized seizures (GS) in PACK and bPAC mice during LFP recordings. **A** Representative spontaneous electrographic GS in a PACK mouse. The hypersynchronous activity spread across both hemispheres and was followed by postictal depression. **B** Similar GS were recorded in bPAC mice. **C**, **D** The number of GS was counted in all LFP recording types (baseline: “ref/pre,” “light ON,” “post”) of PACK and bPAC mice, and the average number of GS in three hours is presented and compared with Friedman’s test followed by Dunn’s multiple comparisons. The occurrence of GS in baseline recordings indicates seizure generalization independent from light-induced PACK/bPAC activation. **E** The total count of GS in LFP recordings (9–28 h) was the highest in bPAC mice. Control mice that expressed either mCherry in CA1 or no virus experienced no seizure-like activity during recordings. Kruskal-Wallis test with Dunn’s multiple comparisons, *p < 0.05, **p < 0.05. Median presented with IQR as error bars. Individual data values are presented in Additional file 3

In healthy control mice, which received intrahippocampal saline and recording electrodes, epileptiform activity is normally absent in the LFP [34]. Unexpectedly, in the majority of the PACK (5 out of 6) and all of the bPAC mice (*n* = 6), hypersynchronous activity, spreading across both hemispheres, arose at least once during LFP recordings (Fig. 5A, B, E). Most of these electrographic generalized seizures were accompanied by behavioral correlates such as freezing, nodding, forelimb clonus, or rearing according to the Racine scale [35].

The occurrence of generalized seizures in baseline recordings (“ref” and “pre”) indicates seizure initiation independent from light-induced PACK (Fig. 5C) and bPAC (Fig. 5D) activation. Illumination of bPAC-expressing neurons increased the median of average seizure count in 3 h compared to post-recordings (“pre” 0.09 [0, 0.48], “light ON” 1.13 [0.45, 3.94], “post” 0 [0, 0.86], Friedman test: *Fr* = 6.38, *n* = 6, *p* = 0.041, Dunn’s post hoc: *p* = 0.042; Fig. 5D). The median number of generalized seizures in all recordings was the highest in bPAC mice (3.50 [1.00, 9.75]; Fig. 5E). Most PACK mice experienced one generalized seizure throughout all the recordings (in total 9–28 h) (1.00 [0.75, 2.75], *n* = 6), whereas mCherry (*n* = 4) and “no virus” (*n* = 7) control mice had no generalized seizures (Fig. 5E). These results suggest that the dark activity of bPAC is responsible for spontaneous generalized seizures arising in mice that express PACK or bPAC in CA1 pyramidal cells.

### Histological abnormalities in PACK- and bPAC-expressing CA1

An optogenetic tool suitable for long-term in vivo experiments should preserve the normal physiology and histology in the target area. For histological analysis, PACK, bPAC, and mCherry mice were perfused after the last LFP recording, 30–35 days after the intrahippocampal virus and saline injections. Coronal sections were immunolabeled with antibodies against neuronal nuclei (NeuN) and glial fibrillary acidic protein (GFAP) to investigate the histology of neurons and astrocytes, respectively.

**Fig. 6:**
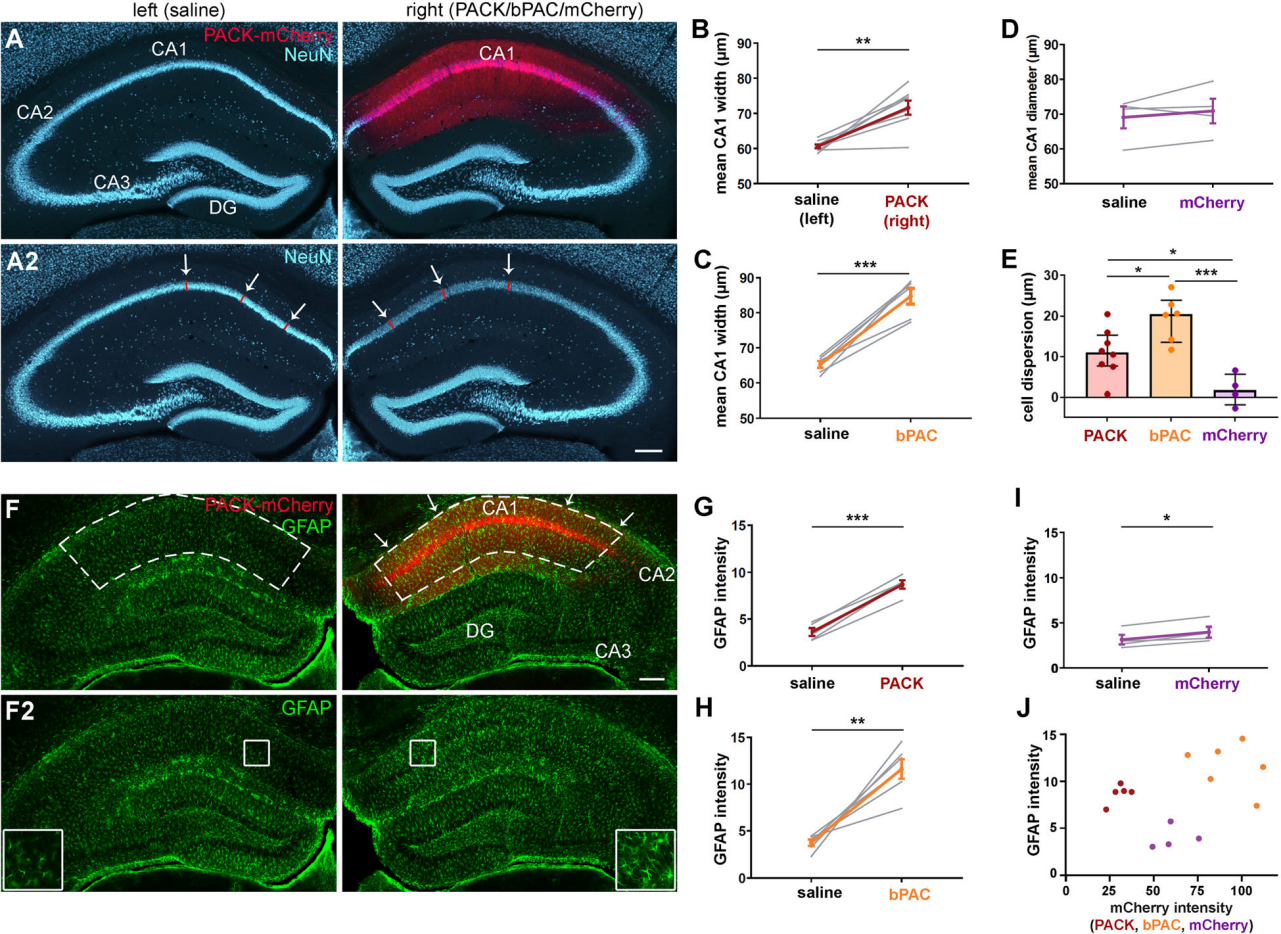
Cell dispersion and astrogliosis in PACK- and bPAC-expressing CA1. **A** Representative image of a NeuN-labeled hippocampal section with PACK-mCherry expression in the right CA1. The diameter of the CA1 pyramidal cell layer was measured at six positions (red lines and white arrows) in three hippocampal sections per animal. **B**, **C** The mean width of the CA1 pyramidal cell layer in **B** PACK-expressing and **C** in bPAC-expressing hippocampus was significantly increased compared to the contralateral saline-injected hippocampus (paired *t*-test). **D** In mCherry-expressing CA1, the pyramidal cell layer width was similar as in the contralateral CA1. **E** The cell dispersion (right-left CA1 width) was the strongest in bPAC mice (one-way ANOVA, Tukey’s post hoc). **F** In the representative image of a GFAP-stained section, GFAP labeling was visibly stronger in the PACK-expressing CA1. Mean gray value of GFAP labeling and mCherry expression was measured in the right dorsal CA1 including *str. oriens*, *str. pyramidale*, and *str. radiatum* (white dashed lines with arrows) in three sections per animal. GFAP intensity was measured at same positions in the left CA1 (white dashed lines). GFAP labeling intensity was significantly higher in **G** PACK, **H** bPAC, and **I** mCherry-expressing CA1 compared to the left saline-injected side (paired *t*-test). **J** There was no clear correlation between GFAP labeling intensity and mCherry intensity; however, PACK, bPAC, and mCherry mice formed three separate clusters with mCherry mice having the lowest GFAP labeling. *p < 0.05, **p < 0.01, ***p < 0.001. Mean presented with SEM as error bars. Scale bars: 200 μm. Individual data values are presented in Additional file 3

To our surprise, we found notable widening of the pyramidal cell layer in PACK-expressing CA1 (Fig. 6A). The mean width of the pyramidal cell layer in PACK-expressing right CA1 was significantly higher than in the left side (right CA1 71.66 ± 2.01 μm, left CA1 60.68 ± 0.54 μm, paired *t*-test: *t* = 5.25, *n* = 8, *p* = 0.0012, Fig. 6B). The pyramidal cell layer was also significantly wider in bPAC-expressing CA1 compared to its contralateral counterpart (right CA1 84.73 ± 2.23 μm, left CA1 65.27 ± 0.94 μm, paired *t*-test: *t* = 8.42, *n* = 6, *p* = 0.0004; Fig. 6C). Mice, which received the same viral vector, carrying just the reporter mCherry, had similar pyramidal cell layer widths in the left and right hippocampus (right CA1 70.96 ± 3.53 μm, left CA1 69.10 ± 3.17 μm, paired *t*-test: *t* = 0.95, *n* = 4, *p* = 0.41; Fig. 6D). Cell dispersion, taken as the difference between right and left CA1 width, was the highest in bPAC-expressing CA1 (19.45 ± 2.31 μm), followed by PACK-expressing CA1 (10.98 ± 1.84 μm), and lacking in mCherry-expressing CA1 (1.86 ± 1.94 μm, one-way ANOVA: *F*(3,18) = 12.51, *p* = 0.0006, Tukey’s multiple comparison test: PACK vs. bPAC *p* = 0.0302, PACK vs. mCherry *p* = 0.040, bPAC vs. mCherry *p* = 0.0005; Fig. 6E). These findings led us to conclude that bPAC, and not the viral vector itself, is inducing the cell dispersion in the CA1 pyramidal cell layer.

GFAP labeling in hippocampal sections shows salient chronic astrogliosis in the PACK-expressing CA1 area (Fig. 6F, F2). The comparison of GFAP labeling in left and right hippocampi revealed strongly elevated GFAP intensity in the PACK-expressing CA1 (right CA1 8.70 ± 0.46, left CA1 3.62 ± 0.41, paired *t*-test: *t* = 13.54, *n* = 5, *p* = 0.0002; Fig. 6G). In bPAC mice, we also found notable astrogliosis in the right bPAC-expressing hippocampus (right CA1 11.62 ± 1.03, left CA1 3.73 ± 0.34, paired *t*-test: *t* = 6.01, *n* = 6, *p* = 0.0018, Fig. 6H). In mCherry mice, the GFAP intensity was slightly but significantly elevated in the right hippocampus (right CA1 3.98 ± 0.61, left CA1 3.15 ± 0.53, paired *t*-test: *t* = 4.12, *n* = 4, *p* = 0.026; Fig. 6I). Thus, it could be that either the viral vector or the presence of an electrode and an optic fiber contributed to the glial scarring in the right hippocampus. There was no significant correlation between the strength of mCherry expression and GFAP intensity in PACK, bPAC, and mCherry mice (Pearson’s correlation: *r* = 0.35, *p* = 0.2; Fig. 6J). However, the three groups formed separate clusters with (1) mCherry mice having medium mCherry expression but the lowest GFAP intensity, (2) PACK mice having the lowest mCherry expression but medium GFAP intensity, and (3) bPAC mice having the strongest mCherry expression and the highest GFAP intensity. Taken together, it seems as if bPAC expression is the main factor inducing chronic astrogliosis in PACK and bPAC mice, while the viral vector and hippocampal implantations might contribute additionally.

### PACK/bPAC expression in the contralateral hippocampus prevents seizure spread in chronically epileptic mice

To find out if the PACK silencer might be useful to limit the spread of epileptiform activity, we targeted PACK to the CA1 principal cells, contralateral to ihpKA treatment. The ihpKA mouse model recapitulates the main pathological features of MTLE, including focal recurrent seizures associated with hippocampal sclerosis [21]. Most ihpKA mice have epileptiform activity that occurs in form of bursts originating in the seizure focus and propagating into the contralateral hippocampus [19, 20, 24]. These epileptiform bursts are subclinical, in other words electrographically measurable but without behavioral convulsions [24, 34]. Unexpectedly, all our PACK-injected ihpKA mice (*n* = 5) were free of contralateral epileptiform bursts already in baseline recordings before light activation of PACK.

**Fig. 7:**
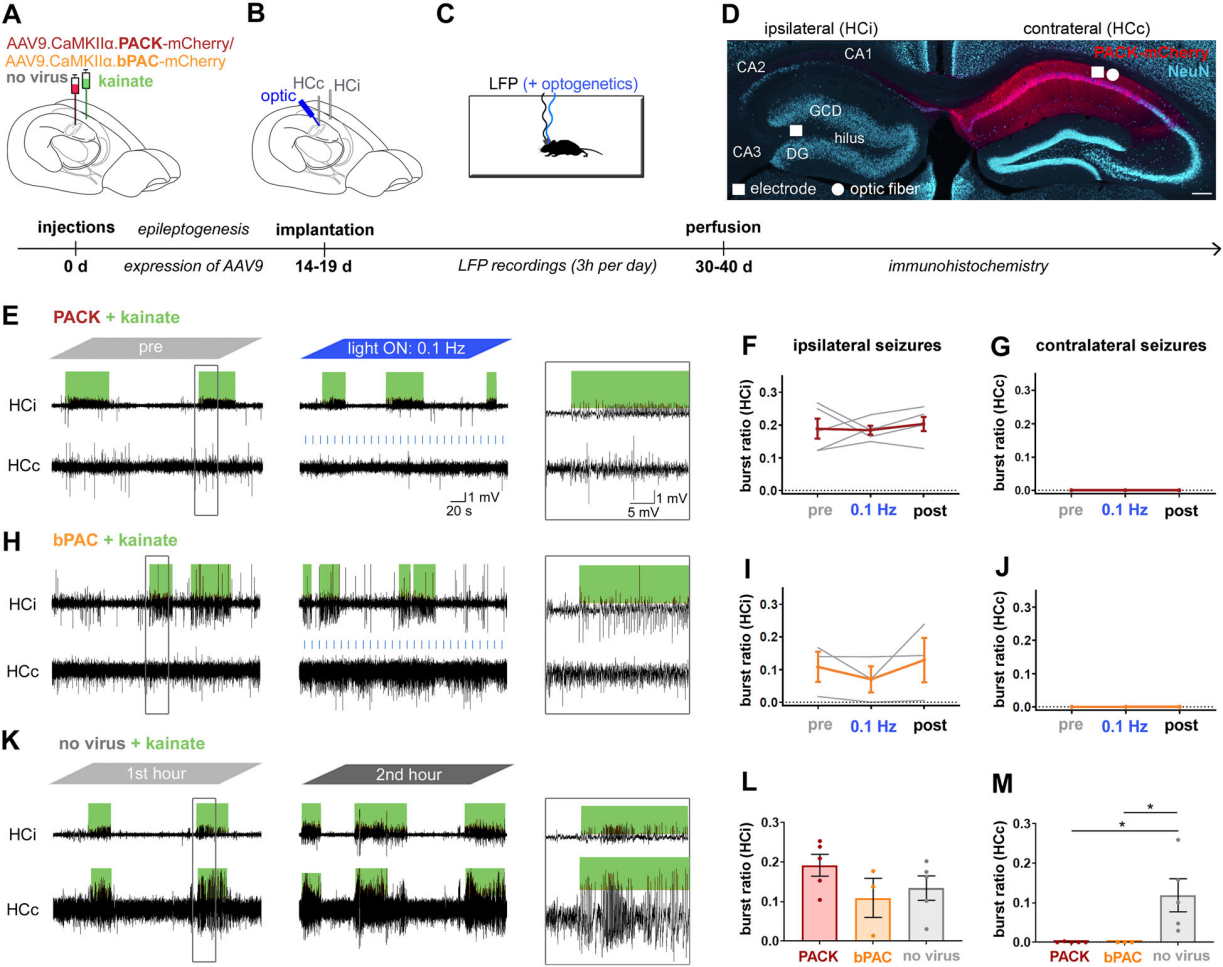
Chronically epileptic PACK and bPAC mice lack epileptiform bursts in the contralateral hippocampus. **A** Mice were injected with kainate in the left hippocampus and AAV9 carrying PACK, bPAC, or no virus in the contralateral hippocampus. **B** Two weeks later, wire electrodes were implanted into the kainate-injected ipsilateral hippocampus (HCi) and the virus-injected contralateral hippocampus (HCc). In PACK and bPAC mice, an optic fiber was implanted at a 30° angle adjacent to the electrode in HCc. **C** Three-hour LFP recordings with and without optogenetic manipulations were performed as previously in healthy PACK/bPAC mice. “No virus” mice were recorded only for 3-h reference recordings. **D** Representative section of an ihpKA PACK mouse showing hippocampal sclerosis in HCi with cell loss in CA1, CA3, and hilus regions as well as granule cell dispersion (GCD). PACK-mCherry was expressed in dorsal CA1 of HCc. Representative LFP snippets from **E** PACK-expressing, **H** bPAC-expressing, and **K** “no virus” kainate-injected mice in the chronic phase of epilepsy. **E**–**M** Spontaneous epileptiform bursts (hypersynchronous spiking activity, marked in green) were detected by an automated algorithm [36], and quantified as burst ratio, a fraction of recording spent in bursts. Each recording type was repeated twice and the mean of burst ratio of the two is presented for each animal. **E**–**G** PACK (*n* = 5) and **H**–**J** bPAC (*n* = 3) ihpKA mice had regularly occurring epileptiform bursts in HCi but no propagation to HCc during pre-recordings as well as during and after illumination. **K** In contrast, ihpKA mice without virus expression (*n* = 5) frequently showed burst propagation to HCc. **L** The burst ratio in HCi was similar in PACK, bPAC, and “no virus” mice, **M** whereas in HCc, the burst ratio was significantly above zero in “no virus” epileptic mice (33–36 days after ihpKA, average burst ratio during a 3-h reference recording). One-way ANOVA with Dunnett’s multiple comparison test, *p < 0.05. Mean presented with SEM as error bars. Individual data values are presented in Additional file 3

We detected the epileptiform bursts with high spike load using a machine learning algorithm [36] and quantified the burst ratio, the fraction of time spent in bursts during the respective recording (Fig. 7). Although the epileptiform bursts occurred frequently in the ipsilateral hippocampus of PACK ihpKA mice (mean burst ratio during “pre” 0.19 ± 0.03, “0.1 Hz” 0.18 ± 0.01, “post” 0.20 ± 0.02, *n* = 5, Fig. 7E-F), the contralateral hippocampus was devoid of epileptiform bursts (Fig. 7G). Similarly, in bPAC ihpKA mice, epileptiform bursts were detected in the ipsilateral (mean burst ratio during “pre” 0.11 ± 0.05, “0.1 Hz” 0.07 ± 0.04, “post” 0.13 ± 0.07, *n* = 3; Fig. 7H-I) but not the contralateral hippocampus (Fig. 7J). Neither the light-induced activation of bPAC nor the whole PACK construct had any effect on the burst ratios in the ipsilateral or contralateral hippocampus (RM ANOVA, *p* > 0.05, Fig. 7E–J).

We compared the burst ratios in ihpKA PACK and bPAC mice to ihpKA “no virus” mice (*n* = 4) in a 3-h recording 33–36 days after kainate to clarify whether the lack of contralateral epileptiform bursts affects the seizure burden in the kainate-injected ipsilateral hippocampus (Fig. 7K–M). There was no significant difference in the ipsilateral burst ratios (PACK 0.19 ± 0.03, bPAC 0.11 ± 0.05, "no virus" 0.13 ± 0.03, one-way ANOVA: *F* = 1.53, *n* = 3–5, *p* = 0.26; Fig. 7L), whereas the mean contralateral burst ratio was evidently higher in “no virus” mice than in PACK and bPAC mice (PACK 0.00 ± 0.00, bPAC 0.00 ± 0.00, "no virus" 0.12 ± 0.04, one-way ANOVA: *F* = 6.26, *n* = 3–5, *p* = 0.017, Dunnett’s multiple comparison test: PACK vs. "no virus" *p* = 0.018, bPAC vs. "no virus" *p* = 0.036; Fig. 7M). These results suggest that the dark activity of bPAC in CA1 pyramidal neurons prevents the spread of epileptiform burst activity to the bPAC-expressing areas. The activity of CA1 pyramidal cells is thus critical for seizure propagation into the contralateral hippocampus. Additionally, the absence of contralateral bursts does not affect the seizure burden in the sclerotic hippocampus.

## Discussion

The present study provides a detailed characterization of a novel two-component potassium channel-based silencer, PACK, and its long-term application in freely moving mice. We targeted the PACK construct to hippocampal CA1 with an AAV9.CaMKIIα vector, resulting in robust expression of PACK-mCherry in principal neurons of this area. We report that PACK is a suitable silencer to reduce the activity of hippocampal neurons in vivo by applying short blue light pulses via an implanted optic fiber. Previously, it was demonstrated in acute hippocampal slices that shining 5-ms blue light pulses at 0.05 Hz onto PACK-expressing CA1 cells was sufficient to abolish spiking elicited by current injections [11]. In our freely moving mice, applying 10-ms light pulses at 0.05 Hz provided an unstable lowering of the LFP magnitude with periods of recovery. However, with an increased illumination frequency of 0.1 Hz, neuronal activity was persistently reduced throughout the 50-min light ON phase. This is a significant improvement compared to microbial chloride and proton pumps, which require continuous illumination and already exhibit declining photocurrent amplitudes within 1 min [1, 37].

Regarding oscillatory activity in CA1, light-induced PACK activation in CA1 pyramidal cells mainly reduced the power of gamma oscillations. Hippocampal gamma rhythm depends on the firing of pyramidal cells and their synchronized dendritic and perisomatic inhibitory input, which originate from local somatostatin- and parvalbumin-positive interneurons, respectively [38, 39]. Constant reduction of pyramidal cell activity consequently decreases local interneuron activity [40], which would explain the lower power of gamma oscillations during PACK-mediated inhibition. PACK-mediated inhibition affected theta oscillations to a much lesser extent, probably because theta rhythm is predominantly driven by GABAergic and cholinergic inputs from the medial septum while depending less on local excitation and inhibition [41–43].

Using soluble bPAC as the light-sensitive domain in the PACK tool is favorable to ensure high light sensitivity [10, 11]. However, this advantage seems to have come with dark activity, which is probably the reason for the side effects we observed in PACK- and bPAC-expressing hippocampi in vivo. The harmful effects of chronic bPAC expression in the hippocampal CA1 area included pyramidal cell layer widening, chronic astrogliosis, and spontaneous generalized seizures. Mice that received the AAV9 vector carrying only mCherry under the CaMKIIα promoter did not show these side effects, except for astrogliosis, which could be due to implantation-related scarring. Based on these findings, we conclude that the viral vector and the fluorescent marker alone were not detrimental. As bPAC mice tended to have higher expression of bPAC-mCherry, more prominent CA1 dispersion, stronger astrogliosis, and higher occurrence of generalized seizures than PACK mice, chronic cAMP elevation may be the underlying cause of these adverse effects.

The second messenger molecule cAMP has several molecular targets in neurons, including protein kinase A (PKA), exchange protein activated by cAMP (Epac), and cAMP-gated ion channels. Through these pathways, cAMP regulates fundamental physiological processes such as growth, metabolism, migration, apoptosis, gene transcription, neurotransmission, and plasticity (for review, see [44–46]). Activation of cAMP-responsive element binding protein (CREB)-mediated gene expression could explain histological changes and seizure activity in bPAC mice as the CREB-transcriptional pathway is involved in acute and chronic phases of epilepsy [29, 47–49]. A study utilizing transgenic mice with constitutively active CREB showed that chronic elevation of CREB activity in hippocampal principal neurons led to increased excitability of CA1 pyramidal neurons, significant loss of hippocampal neurons, and sporadic epileptic seizures [50], resembling what we observed in our bPAC mice.

Light activation of bPAC in vivo strongly increased the LFP magnitude in the CA1 region with mainly augmenting beta and gamma oscillations. The neuronal activity dropped immediately after the illumination phase, suggesting a temporary depolarizing effect via a cyclic nucleotide-gated channel or induction of short-term potentiation. Local cAMP elevation induces presynaptic potentiation by promoting the accumulation of calcium channels close to release sites, thus increasing the release probability [31, 51, 52]. Presynaptic elevation of cAMP via light activation of synapse-targeted bPAC was sufficient to trigger potentiation at the mossy fiber-CA3 synapse but not in the CA3-CA1 synapse [53]. Although cAMP-mediated presynaptic potentiation is not exhibited by all hippocampal synapses, it is present in the CA1-subicular bursting neuron synapse [54]. Therefore, temporarily increased transmission at the CA1-subiculum synapses may have contributed to light-induced hyperactivity in bPAC-expressing CA1 in vivo. Alternatively, cAMP binding on hyperpolarization-activated cyclic nucleotide-gated (HCN) cation channels might have increased pyramidal cell excitability because (1) HCN channels are abundantly expressed in hippocampal neurons and (2) the inward currents through HCN channels depolarize the membrane [55–57]. Furthermore, HCN channels are thought to initiate rhythmic firing [57, 58], which could theoretically explain elevated beta and gamma oscillations during light-induced bPAC activation in vivo. Future experiments with probes or tetrodes could potentially clarify the mechanism of bPAC-mediated excitation and PACK-mediated inhibition at a single-unit level.

In the last part of our study, we applied the PACK silencer in chronically epileptic ihpKA mice, which usually exhibit spontaneous seizures in both hippocampi [19, 20, 24]. We targeted PACK to the contralateral CA1 area (opposite to kainate injection) to determine if we can interfere with seizure spread between the two hippocampi. To our surprise, there was no propagation of seizure activity from the kainate-injected hippocampus to the PACK-expressing contralateral side, even before light activation of PACK. We saw the same in bPAC mice, which were also lacking contralateral seizures already in the baseline recordings. Surprisingly, prolonged bPAC expression in CA1 pyramidal cells induced generalized seizures in both saline- and kainate-injected mice, whereas it prevented the spread of subclinical seizures in kainate-injected mice. The role of cAMP in epileptiform activity has also shown to be contradictory in previous studies using cAMP analogs or forskolin, an activator of adenylyl cyclase. For example, in an in vitro study, forskolin increased the epileptiform bursting activity induced by electrical stimulation of the corpus callosum [59]. On the other hand, systemic injection of forskolin before seizure induction with pentylenetetrazol prevented tonic seizures in mice [60]. Hypothetically, bPAC-dependent cAMP elevation and subsequent HCN channel activation could explain contrasting findings regarding epileptogenicity, since HCN channels have also been found to act pro- or anti-epileptic [61–64]. Future work comparing gene expression and hippocampal slice electrophysiology in saline- and kainate-treated mice with and without bPAC expression would be needed to address the mechanism of this cell-specific cAMP-associated seizure induction and prevention.

Our results suggest that the expression of bPAC affects the physiology of hippocampal principal cells in the absence of blue light. Functional dark activity of soluble bPAC has also been reported by others, who utilized a fluorescent PKA sensor to detect intracellular cAMP levels in bPAC-expressing CA1 cells in hippocampal slice cultures [65]. For reduced dark activity, the soluble bPAC from the *Beggiatoa* bacterium could be replaced by another photoactivated adenylyl cyclase (PAC). There are several PACs available from other microorganisms, which have lower light sensitivity but still provide sufficient potassium current when coupled to SthK [11]. Alternatively, a red-shifted bPAC with reduced dark activity could be used [66]. Another approach would be targeting a PAC to the cell membrane. For instance, a recently developed membrane-anchored PAC has no detectable dark activity in hippocampal neurons [65]. Similarly, membrane-bound guanylyl cyclase rhodopsins, which have mutated to be adenylyl cyclases, virtually lack dark activity [67, 68]. In these approaches, the possibility of SthK channel activation by intrinsic cAMP remains. Accordingly, expression of SthK without bPAC in body wall muscle cells of *C. elegans* was already sufficient to see a behavioral change resulting from muscle hyperpolarization [68]. This problem could be overcome by engineering SthK variants with mutations in the cAMP binding site, resulting in a channel with reduced cAMP affinity.

## Conclusion

Taken together, we showed in awake mice that the potassium channel-based optogenetic silencer PACK reliably reduces hippocampal neuronal activity in a light-dependent manner. In contrast to other optogenetic inhibitors, PACK requires only short light pulses at a low frequency to achieve a prolonged reduction of neuronal activity. A disadvantage of PACK is its light-active component, bPAC, since it elicits side effects in vivo, which are presumably related to its dark activity. In the mouse model of MTLE, the light-independent effects of bPAC prevented the spread of spontaneous epileptiform activity from the seizure focus to the contralateral bPAC-expressing CA1 region. Our study underlines that the PACK tool is a potent optogenetic inhibitor but refinement of its light-sensitive domain is required to avoid dark activity and related side effects.

## Methods

### Animals

Experiments were performed in adult 10- to 21-week-old transgenic male mice (C57BL/6-Tg(Thy1-eGFP)-M-Line, own breeding) [69]. For this study, 62 mice were used, which were randomly allocated to one of the experimental groups (Table 1). Mice were kept in a 12-h light/dark cycle at room temperature (RT) with food and water ad libitum. All animal procedures were carried out following the guidelines of the European Community’s Council Directive of 22 September 2010 (2010/63/EU) and were approved by the regional council (Regierungspräsidium Freiburg).

**Table 1:** Experimental groups and sample sizes. From the initial number of mice that entered the experiment (total injected), some died owing to intrahippocampal kainate injections, anesthesia, or implantations. *Some mice were excluded from the LFP analysis due to electrode/optic fiber positions not in CA1 (*n* = 4), due to lack of signal from the electrode (*n* = 1), or due to unusual hippocampal atrophy (*n* = 2). **Two mice were excluded from histological analysis because of widespread PACK-mCherry expression not only in CA1 but also in DG. ***Three mice were further excluded from the analysis of GFAP intensity because of incomparable labeling intensity. The final sample sizes for LFP analysis and histological analysis are presented in the last two columns

Group	Total injected	Died due to surgery	Excluded*/**	LFP analysis	Histological analysis
Saline + PACK	11	2	4*, 2**	6	8/5***
Saline + bPAC	8	1	1*,1**	6	6
Saline + mCherry	4	1	1*	3	4
Saline + no virus	9	2	0	7	–
Kainate + PACK	11	4	2*	5	–
Kainate + bPAC	9	6	0	3	–
Kainate + no virus	10	5	0	5	–

### Stereotaxic intrahippocampal injections

Mice were stereotaxically injected with a recombinant adeno-associated viral (AAV) construct into the right hippocampus and either with saline or kainate into the left hippocampus (see experimental groups in Table 1). These injections were performed in one surgery under deep anesthesia (ketamine hydrochloride 100 mg/kg, xylazine 5 mg/kg, atropine 0.1 mg/kg body weight, i.p.) [23, 70, 71] using Nanoject III (Drummond Scientific Company, Broomall, Pennsylvania, USA). AAVs were injected into the right dorsal stratum pyramidale of CA1 (stereotaxic coordinates relative to bregma: anterioposterior [AP] = −2.0 mm, mediolateral [ML] = −1.3 mm and relative to cortical surface: dorsoventral [DV] = −1.15 mm). Each mouse received an injection of either 0.9% sterile saline or 15 mM kainate (50 nL, Tocris, Bristol, UK) into the left stratum lacunosum-moleculare of CA1 (AP = −2.0 mm, ML = + 1.5 mm, DV = −1.5 mm).

For optogenetic inhibition of CA1 principal cells, the injected AAV was carrying the PACK silencer with a red fluorescent marker mCherry under the control of the Ca^2+^/calmodulin-dependent kinase II alpha (CaMKIIα) promoter (AAV9.CamKIIα:SthK-P2A-bPAC-mCherry referred to as AAV9.CaMKIIα:PACK-mCherry). In order to attribute the effects of AAV9.CaMKIIα:PACK-mCherry to its components, we included three control groups, in which mice received (1) a construct carrying the adenylyl cyclase bPAC but lacking the SthK channel (AAV9.CamKIIα:bPAC-mCherry), (2) a construct carrying only mCherry (AAV9.CamKIIα:mCherry), or (3) no virus (for sample sizes, see Table 1). The volume of the injected virus was adjusted to achieve an optimal and comparable expression pattern in CA1: (1) PACK—300 nL, (2) bPAC—250–300 nL, and (3) mCherry—300 nL with 1:5 dilution. All viral constructs were obtained from the Viral Core Facility, *Charité* – Universitätsmedizin *Berlin*, *Germany.*

In those mice that received kainate, the occurrence of a behavioral *status epilepticus* was verified by observation of mild convulsion, chewing, immobility, or rotations, as described before [22, 72]. Fifteen mice died as a consequence of status epilepticus and further two were excluded due to extreme hippocampal atrophy (Table 1).

### Electrode and optic fiber implantations

Implantations were performed 14–19 days after intrahippocampal injections as described previously [24]. For LFP analysis, we implanted a Teflon-coated platinum-iridium wire electrode (125-μm diameter; World Precision Instruments, Sarasota, FL, USA) into each hippocampus: AAV-injected right CA1 (AP = − 2.0 mm, ML = −1.4 mm, DV = − 1.1 mm) and saline/kainate-injected left DG (AP = − 2.0 mm, ML = + 1.4 mm, DV = − 1.6 mm). An optic fiber (ferrule 1.25 mm, cannula 200-μm diameters; Thorlabs Inc., Newton, NJ, USA) was implanted into the AAV-injected CA1, adjacent to the electrode at a 30° angle (AP = − 2.0 mm, ML = − 2.4 mm, DV = − 1.0 mm). Two stainless steel screws (DIN 84; Schrauben-Jäger, Landsberg, Germany) were implanted above the frontal cortex to provide a reference and ground. Electrodes and screws were soldered to a micro-connector (BLR1-type) and fixed with dental cement (Paladur, Kulzer GmbH, Hanau, Germany). The electrode and optic fiber positions were confirmed by *post hoc *histology (Additional file 1: Fig. S1). Five mice were excluded from LFP analysis due to electrode/optic fiber locations in the cortex above CA1. Three mice died following implantation procedures (Table 1).

### Electrophysiological recordings and optogenetic manipulations

Three-hour-long LFPs were acquired from freely moving mice in the period of 19–40 days after intrahippocampal injections. For LFP recordings, mice were connected to a miniature preamplifier (MPA8i, Smart Ephys/Multi Channel Systems, Reutlingen, Germany). Signals were amplified 1000-fold, bandpass-filtered from 1 Hz to 5 kHz and digitized with a sampling rate of 10 kHz (Power1401 analog-to-digital converter, Spike2 software, Cambridge Electronic Design, Cambridge, UK).

For each AAV9-injected mouse (with PACK, bPAC or mCherry), we acquired reference LFPs before, in between, and after illumination experiments. LFPs with 1-h illumination at 0.05 Hz or 0.1 Hz were recorded twice per frequency on separate days. Each mouse represents a biological replicate (*n* = 3–7 per group, Table 1) and the number of recordings per mouse a technical replicate (*n* = 2 per recording type). We used the biological replicate as our sample size in statistical testing and presented the average of the two sessions since there was no significant difference between the recording replications (Additional file 1: Fig. S4).

The sessions with illumination comprised 1-h pre-recording, “light ON” phase during the second hour and post-recording during the third hour. During the “light ON” phase, blue light pulses (460 nm, ~ 80 mW/mm^2^, 10-ms pulse duration, blue LED from Prizmatix Ltd. Givat-Shmuel, Israel) were applied every 10 or 20 s. To hinder rebound excitation resulting from the illumination off-set [11, 73], each light pulse had a 5-ms ramp-like termination (within pulse fade-off). Furthermore, during the last 10 min of the “light ON” phase, 5-ms light pulses were applied with gradually reducing intensity (within recording fade-off). The light pulse duration and frequencies were selected based on previous work by Bernal Sierra et al., who demonstrated that shining 5-ms light pulses with 0.05 Hz in hippocampal slices provided long-lasting stable inhibition of current-elicited spiking in PACK-expressing CA1 pyramidal cells [11].

Kainate-injected mice, which were implanted with intrahippocampal electrodes but did not receive a viral vector or an optic fiber, served as epileptic “no virus” controls. Three-hour recordings from these mice were performed on day 35 or 36 after kainate injection.

### Analysis of local field potentials

LFP data were visually inspected with Spike2 software and analyzed in detail using Python 2.7. The line length, a sum of distances between successive data points, was selected as a measure of the LFP waveform dimensionality since it is sensitive to variations in both amplitude and frequency [74]. We calculated the line length of downsampled data (500 Hz) by using the following equation, where *L* is the line length (mV/s), *x* is the data-trace, *k* is one data point, and *t* is the recording duration in seconds:

$$ L=\frac{\sum_{k=1}^N abs\left[x\left(k-1\right)-x(k)\right]}{t} $$

The line lengths were calculated for the first, second, and third hour of each recording. In recordings with illumination, the line length calculations and spectral analysis were done on LFP data recorded during the first 50 min; the last 10 min was omitted due to light intensity fade-off. Furthermore, in recordings with illumination, the line lengths shortly after the light pulses were compared to the line lengths directly before the light pulses. For this, 2-s snippets were extracted before and after each light pulse during the first 50 min of the “light ON” phase. In addition, 2-s snippets at corresponding time points were extracted in pre-recordings. Subsequently, the mean “pre,” “before pulse,” and “after pulse” line lengths were calculated for each recording session (2 per animal) and an average line length was presented for all animals. Periods with electrographic generalized seizures (GS), where hypersynchronous neuronal activity was propagating across hemispheres followed by postictal depression, were removed from analysis due to strongly altered LFP characteristics.

The frequency compositions of the LFPs are presented with PSDs and spectrograms generated by fast Fourier transform (FFT) of LFP raw data (sampling rate 10 kHz). The PSDs were estimated by applying Welch’s method in Python 2.7 (scipy.signal.welch function), based on time averaging over short periodograms (periodogram length = 10x sampling rate). The oscillatory power was calculated as the area under the PSD plot for the respective frequency ranges: delta (1–4 Hz), theta (4–12 Hz), beta (12–30 Hz), and gamma (30–120 Hz). For time-frequency representation of the LFP power in a spectrogram, the Hanning window was applied and data in the time domain (length of FFT = 10x sampling rate) was broken up into overlapping (overlap = 0.25x sampling rate) segments (segment length = 0.5x sampling rate).

### Analysis of epileptiform activity

Downsampled hippocampal LFPs recorded from epileptic animals were analyzed in detail using a custom-made semi-automated algorithm that detects and classifies epileptiform activity [36]. In the ihpKA mouse model, epileptiform activity occurs as single sharp wave epileptiform spikes and as bursts, which are clusters of many spikes [22]. The algorithm classifies the bursts according to their spike load into low-load, medium-load, and high-load bursts as described by Heining et al. [36]. To assess the effect of PACK-mediated inhibition on seizure activity, we calculated the “burst ratio,” which is the duration of high-load bursts per total recording time. The automatic detection of high-load bursts was verified by visual inspection of the LFP recordings. Sessions with GS were removed from the analysis due to long-lasting suppression of neuronal activity after such a seizure.

### Perfusion and tissue preparation

After the last LFP recording (at day 30–40), the mice were deeply anesthetized and transcardially perfused with 0.9% saline followed by 4% paraformaldehyde in 0.1 M phosphate buffer (PB, pH 7.4). The brains were dissected and post-fixated overnight in 4% paraformaldehyde, followed by sectioning (coronal plane, 50 μm) with a vibratome (VT100S, Leica Biosystems, Wetzlar, Germany). The slices were collected and stored in PB for immunohistochemistry.

### Immunohistochemistry

To determine optic fiber and electrode positions, to visualize the hippocampal anatomy after AAV9 injections, and to validate hippocampal sclerosis in ihpKA mice, we performed immunohistochemistry with markers for neurons and astrocytes. For the immunofluorescence staining, free-floating sections were pre-treated with 0.25% TritonX-100 and 10% normal horse or goat serum (Vectorlabs, Burlingame, CA, USA) diluted in PB for 1 h. Subsequently, slices were incubated either with guinea-pig anti-NeuN (1:500; Synaptic Systems, Göttingen, Germany, 266004, RRID:AB_2713971) or rabbit anti-GFAP (1:500, Dako, Glostrup, Denmark, Z0334, RRID:AB_10013382) overnight at 4 °C. Sections were rinsed and then incubated for 2.5 h in donkey anti-guinea-pig or donkey anti-rabbit Cy5-conjugated secondary antibody (1:200, Jackson ImmunoResearch Laboratories Inc., West Grove, PA, USA, anti-guinea-pig: 706-175-148, RRID:AB_2340462; anti-rabbit: 711-175-152, RRID:AB_2340607), followed by extensive rinsing in PB. The sections were mounted on glass slides and coverslipped with Immu-Mount^TM^ mounting medium (Thermo Shandon Ltd, Runcorn, UK).

### Image acquisition and histological analysis

Tiled fluorescent images of the brain sections were taken with an *AxioImager 2* microscope (Carl Zeiss Microscopy GmbH, Jena, Germany) using a Plan-Apochromat 10x objective with numerical aperture 0.45 (Zeiss, Göttingen, Germany). The exposure times (Cy5-labeled NeuN 5 s, Cy5-labeled GFAP 3 s, mCherry 300 ms) were kept constant for each staining to allow for comparisons across animals.

To assess the effect of AAV9-mediated delivery of PACK, bPAC, and/or mCherry and long-term expression of these proteins on hippocampal histology, we measured the relative expression intensities of GFAP labeling in the mCherry-expressing dorsal CA1 at three positions along the anteroposterior axis (−1.70 mm, −1.94 mm, and −2.18 mm from bregma). The quantification was performed in Fiji ImageJ by drawing a polygon-shaped region of interest (ROI) around mCherry expression in CA1 (*str. oriens* to *str. radiatum*) and taking the mean gray area of both mCherry and the GFAP labeling within this ROI. Areas with glial scarring around the implantations were excluded by adjusting the ROI. The same measurement was done in the contralateral CA1 by drawing a similar ROI, which avoided implant-related scars. For normalization, in each slice, the local background was measured in a small square (41457 μm^2^) in the cortex and subtracted from the mean gray areas of each ROI in CA1. Finally, mean expression intensities of mCherry and GFAP in left (saline-injected) and right (virus-injected) CA1 were presented for each animal. Furthermore, we quantified the width of the pyramidal cell layer in dorsal medial CA1 by measuring three perpendicular lines in the left and in the right CA1 of each NeuN-labeled section (3 sections per mouse, AP −1.70 mm, −1.94 mm, −2.18 mm from bregma) and compared the mean width of the left and right CA1 pyramidal cell layer.

In ihpKA mice, the presence of hippocampal sclerosis in the kainate-injected hippocampus was confirmed in NeuN-labeled sections showing granule cell dispersion and cell loss in CA1 and CA3 and in GFAP-labeled sections demonstrating astrogliosis.

### Statistical analysis

Data were tested for statistical significance with GraphPad Prism 8 software (GraphPad Software Inc.). To determine how the line length and spectral power changed during the second and third hour of LFP recordings, the baseline value (in the first hour) was subtracted from the original values and tested for significance using a one-sample *t*-test with a Bonferroni correction of the significance level (*α* = 0.025 for two comparisons). A paired *t*-test was used for comparing two matched groups of parametric data (normally distributed with equal variance). Comparisons of more than two parametric data sets were performed either with a one-way ANOVA or with repeated-measures (RM) ANOVA, in case of matched groups. If an ANOVA indicated that not all group means were equal, Tukey’s multiple comparison test was performed additionally. Friedman’s test (matched) or Kruskal-Wallis test (non-matched) with Dunn’s post hoc was applied for comparing three groups of non-parametric data. For determining whether the power of oscillations in different frequency bands changed significantly during the 3-h recordings, two-way RM ANOVA with matching was performed, followed by Dunnett’s multiple comparison test. Pearson’s correlation coefficient was used to measure the strength of association between two variables. Significance thresholds were set to *p < 0.05, **p < 0.01, and ***p < 0.001 (two-tailed *p*-values). For parametric data, mean and SEM are given; for non-parametric data, median with interquartile range (IQR) are reported.

## Supplementary Information

**Additional file 1: Fig. S1.** [Electrode and optic fiber positions in dorsal hippocampi of PACK mice]. **Fig. S2.** [Light application in mCherry mice does not alter neuronal activity in CA1]. **Fig. S3.** [Line length and gamma power decrease during reference recordings in bPAC mice]. **Fig. S4**- [The line length drops similarly in the first and second reference recording].

**Additional file 2: Table S1.** [Results of the spectral analysis in saline PACK mice]. **Table S2**. [Results of the spectral analysis in saline bPAC mice].

**Additional file 3.** Spreadsheets of numerical data in Figs. 5, 6, 7 and Fig. S2.

## Data Availability

All data generated or analyzed during this study are included in this published article, its supplementary information files, and publicly available repositories. The LFP datasets and Python scripts are available on G-Node (10.12751/g-node.ttyt3c). The source code for detection and classification of epileptiform activity is accessible at Zenodo (10.5281/zenodo.4110614) [75]. The viral constructs used in this study are available on request from P. Kleis or from the Viral Core Facility, Charité – Universitätsmedizin Berlin.

## Abbreviations

AAV: Adeno-associated virus
Arc: Activity-related cytoskeleton
AP: Anterioposterior
AUC: Area under the curve
bPAC: Photoactivated adenylyl cyclase from *Beggiatoa*
CA: Cornu ammonis
CaMKIIα: Calcium/calmodulin-dependent protein kinase II α
CREB: cAMP-responsive element binding protein
cAMP: cyclic adenosine monophosphate
DV: Dorsoventral
Epac: Exchange protein activated by cAMP
FFT: Fast Fourier transform
GABA_A_: γ-Aminobutyric acid A
GFAP: Glial fibrillary acidic protein
HCN: Hyperpolarization-activated cyclic nucleotide-gated
ihpKA: Intrahippocampal kainate
IQR: Interquartile range
K^+^: Potassium ions
LFP: Local field potential
ML: Mediolateral
MTLE: Mesial temporal lobe epilepsy
NeuN: Neuronal nuclei
PACK: Optogenetic silencer comprising of bPAC and SthK
PB: Phosphate buffer
PKA: Protein kinase A
PSD: Power spectral density
RM: Repeated measures
ROI: Region of interest
RT: Room temperature
SthK: Potassium channel from *Spirochaeta thermophilia*

## References

[1] Wiegert JS, Mahn M, Prigge M, Printz Y, Yizhar O (2017). Silencing neurons: tools, applications, and experimental constraints. Neuron..

[2] Owen SF, Liu MH, Kreitzer AC. Thermal constraints on in vivo optogenetic manipulations. Nat Neurosci. 22(7):1061–5. 10.1038/s41593-019-0422-3. 10.1038/s41593-019-0422-3 PMC6592769 31209378

[3] Raimondo JV, Kay L, Ellender TJ, Akerman CJ (2012). Optogenetic silencing strategies differ in their effects on inhibitory synaptic transmission. Nat Neurosci..

[4] Alfonsa H, Merricks EM, Codadu NK, Cunningham MO, Deisseroth K, Racca C, Trevelyan AJ (2015). The contribution of raised intraneuronal chloride to epileptic network activity. J Neurosci..

[5] Sørensen AT, Ledri M, Melis M, Ledri LN, Andersson M, Kokaia M. Altered chloride homeostasis decreases the action potential threshold and increases hyperexcitability in hippocampal neurons. eNeuro. 2017;4(6):ENEURO.0172-17.2017. 10.1523/ENEURO.0172-17.2017. 10.1523/ENEURO.0172-17.2017 PMC5783240 29379872

[6] Banghart M, Borges K, Isacoff E, Trauner D, Kramer RH (2004). Light-activated ion channels for remote control of neuronal firing. Nat Neurosci..

[7] Janovjak H, Szobota S, Wyart C, Trauner D, Isacoff EY (2010). A light-gated, potassium-selective glutamate receptor for the optical inhibition of neuronal firing. Nat Neurosci..

[8] Kang JY, Kawaguchi D, Coin I, Xiang Z, O’Leary DDM, Slesinger PA, Wang L (2013). In vivo expression of a light-activatable potassium channel using unnatural amino acids. Neuron..

[9] Cosentino C, Alberio L, Gazzarrini S, Aquila M, Romano E, Cermenati S, Zuccolini P, Petersen J, Beltrame M, van Etten JL, Christie JM, Thiel G, Moroni A (2015). Optogenetics. Engineering of a light-gated potassium channel. Science.

[10] Beck S, Yu-Strzelczyk J, Pauls D, Constantin OM, Gee CE, Ehmann N, Kittel RJ, Nagel G, Gao S (2018). Synthetic light-activated ion channels for optogenetic activation and inhibition. Front Neurosci..

[11] Bernal Sierra YA, Rost BR, Pofahl M, Fernandes AM, Kopton RA, Moser S, Holtkamp D, Masala N, Beed P, Tukker JJ, Oldani S, Bönigk W, Kohl P, Baier H, Schneider-Warme F, Hegemann P, Beck H, Seifert R, Schmitz D (2018). Potassium channel-based optogenetic silencing. Nat Commun..

[12] Stierl M, Stumpf P, Udwari D, Gueta R, Hagedorn R, Losi A, Gärtner W, Petereit L, Efetova M, Schwarzel M, Oertner TG, Nagel G, Hegemann P (2011). Light modulation of cellular cAMP by a small bacterial photoactivated adenylyl cyclase, bPAC, of the soil bacterium Beggiatoa. J Biol Chem..

[13] Brams M, Kusch J, Spurny R, Benndorf K, Ulens C (2014). Family of prokaryote cyclic nucleotide-modulated ion channels. Proc Natl Acad Sci USA..

[14] Engel J Jr. Mesial temporal lobe epilepsy: what have we learned. Neuroscientist. 2001;7(4):340–52. 10.1177/107385840100700410. 10.1177/107385840100700410 11488399

[15] Kwan P, Sander JW (2004). The natural history of epilepsy: an epidemioloqical view. J Neurol Neurosurg Psychiatry..

[16] Gloor P, Salanova V, Olivier A, Quesney LF (1993). The human dorsal hippocampal commissure: an anatomically identifiable and functional pathway. Brain..

[17] Popovic L, Vojvodic N, Ristic AJ, Bascarevic V, Sokic D, Kostic VS (2012). Ictal dystonia and secondary generalization in temporal lobe seizures: a video-EEG study. Epilepsy Behav..

[18] Mintzer S, Cendes F, Soss J, Andermann F, Engel J, Dubeau F, Olivier A, Fried I (2004). Unilateral hippocampal sclerosis with contralateral temporal scalp ictal onset. Epilepsia..

[19] Meier R, Häussler U, Aertsen A, Deransart C, Depaulis A, Egert U (2007). Short-term changes in bilateral hippocampal coherence precede epileptiform events. Neuroimage..

[20] Paschen E, Elgueta C, Heining K, Vieira DM, Kleis P, Orcinha C, Häussler U, Bartos M, Egert U, Janz P, Haas CA (2020). Hippocampal low-frequency stimulation prevents seizure generation in a mouse model of mesial temporal lobe epilepsy. Elife..

[21] Bouilleret V, Ridoux V, Depaulis A, Marescaux C, Nehlig A, Le Gal La Salle G (1999). Recurrent seizures and hippocampal sclerosis following intrahippocampal kainate injection in adult mice: electroencephalography, histopathology and synaptic reorganization similar to mesial temporal lobe epilepsy. Neuroscience..

[22] Riban V, Bouilleret V, Pham-Lê BT, Fritschy JM, Marescaux C, Depaulis A (2002). Evolution of hippocampal epileptic activity during the development of hippocampal sclerosis in a mouse model of temporal lobe epilepsy. Neuroscience..

[23] Janz P, Savanthrapadian S, Häussler U, Kilias A, Nestel S, Kretz O, Kirsch M, Bartos M, Egert U, Haas CA (2017). Synaptic remodeling of entorhinal input contributes to an aberrant hippocampal network in temporal lobe epilepsy. Cereb Cortex..

[24] Janz P, Hauser P, Heining K, Nestel S, Kirsch M, Egert U, Haas CA (2018). Position- and time-dependent arc expression links neuronal activity to synaptic plasticity during epileptogenesis. Front Cell Neurosci..

[25] Buzsáki G, Buhl DL, Harris KD, Csicsvari J, Czéh B, Morozov A (2003). Hippocampal network patterns of activity in the mouse. Neuroscience..

[26] Colgin LL (2016). Rhythms of the hippocampal network. Nat Rev Neurosci..

[27] Rangel LM, Rueckemann JW, Riviere PD, Keefe KR, Porter BS, Heimbuch IS, Budlong CH, Eichenbaum H (2016). Rhythmic coordination of hippocampal neurons during associative memory processing. Elife..

[28] Weisskopf MG, Castillo PE, Zalutsky RA, Nicoll RA (1994). Mediation of hippocampal mossy fiber long-term potentiation by cyclic AMP. Science..

[29] Huang YY, Li XC, Kandel ER (1994). cAMP contributes to mossy fiber LTP by initiating both a covalently mediated early phase and macromolecular synthesis-dependent late phase. Cell..

[30] Gruart A, Benito E, Delgado-García JM, Barco A (2012). Enhanced cAMP response element-binding protein activity increases neuronal excitability, hippocampal long-term potentiation, and classical eyeblink conditioning in alert behaving mice. J Neurosci..

[31] Fukaya R, Maglione M, Sigrist SJ, Sakaba T (2021). Rapid Ca2+ channel accumulation contributes to cAMP-mediated increase in transmission at hippocampal mossy fiber synapses. Proc Natl Acad Sci U S A..

[32] Cabib S, Algeri S, Perego C, Puglisi-Allegra S (1990). Behavioral and biochemical changes monitored in two inbred strains of mice during exploration of an unfamiliar environment. Physiol Behav..

[33] Hinman JR, Penley SC, Long LL, Escabí MA, Chrobak JJ (2011). Septotemporal variation in dynamics of theta: speed and habituation. J Neurophysiol..

[34] Twele F, Schidlitzki A, Töllner K, Löscher W (2017). The intrahippocampal kainate mouse model of mesial temporal lobe epilepsy: lack of electrographic seizure-like events in sham controls. Epilepsia Open..

[35] Racine RJ (1972). Modification of seizure activity by electrical stimulation. II. Motor seizure. Electroencephalogr Clin Neurophysiol.

[36] Heining K, Kilias A, Janz P, Häussler U, Kumar A, Haas CA, et al. Bursts with high and low load of epileptiform spikes show context-dependent correlations in epileptic mice. eNeuro. 2019;6(5): ENEURO.0299-18.2019.10.1523/ENEURO.0299-18.2019. 10.1523/ENEURO.0299-18.2019 PMC6731539 31420348

[37] Mattis J, Tye KM, Ferenczi EA, Ramakrishnan C, O’Shea DJ, Prakash R (2012). Principles for applying optogenetic tools derived from direct comparative analysis of microbial opsins. Nat Methods..

[38] Tukker JJ, Fuentealba P, Hartwich K, Somogyi P, Klausberger T (2007). Cell type-specific tuning of hippocampal interneuron firing during gamma oscillations in vivo. J Neurosci..

[39] Antonoudiou P, Tan YL, Kontou G, Louise Upton A, Mann EO (2020). Parvalbumin and somatostatin interneurons contribute to the generation of hippocampal gamma oscillations. J Neurosci..

[40] Gridchyn I, Schoenenberger P, O’neill J, Csicsvari J (2020). Optogenetic inhibition-mediated activity-dependent modification of CA1 pyramidal-interneuron connections during behavior. Elife.

[41] Hangya B, Borhegyi Z, Szilágyi N, Freund TF, Varga V (2009). GABAergic neurons of the medial septum lead the hippocampal network during theta activity. J Neurosci..

[42] Fuhrmann F, Justus D, Sosulina L, Kaneko H, Beutel T, Friedrichs D, Schoch S, Schwarz MK, Fuhrmann M, Remy S (2015). Locomotion, theta oscillations, and the speed-correlated firing of hippocampal neurons are controlled by a medial septal glutamatergic circuit. Neuron..

[43] Müller C, Remy S (2018). Septo – hippocampal interaction. Cell Tissue Res..

[44] Nguyen PV, Woo NH. Regulation of hippocampal synaptic plasticity by cyclic AMP-dependent protein kinases. Prog Neurobiol. 2003;71(6):401–37. 10.1016/j.pneurobio.2003.12.003. 10.1016/j.pneurobio.2003.12.003 15013227

[45] Cheng X, Ji Z, Tsalkova T, Mei F. Epac and PKA: a tale of two intracellular cAMP receptors. Acta Biochim Biophys Sin (Shanghai). 2008;40(7):651–62. 10.1111/j.1745-7270.2008.00438.x. 10.1111/j.1745-7270.2008.00438.x PMC2630796 18604457

[46] Antoni FA (2012). New paradigms in cAMP signalling. Mol Cell Endocrinol.

[47] Hansen KF, Sakamoto K, Pelz C, Impey S, Obrietan K (2014). Profiling status epilepticus-induced changes in hippocampal RNA expression using high-throughput RNA sequencing. Sci Rep..

[48] Choi YS, Lee B, Hansen KF, Aten S, Horning P, Wheaton KL, Impey S, Hoyt KR, Obrietan K (2016). Status epilepticus stimulates NDEL1 expression via the CREB/CRE pathway in the adult mouse brain. Neuroscience.

[49] Conte G, Parras A, Alves M, Ollà I, De Diego-Garcia L, Beamer E (2020). High concordance between hippocampal transcriptome of the mouse intra-amygdala kainic acid model and human temporal lobe epilepsy. Epilepsia..

[50] De Armentia ML, Jancic D, Olivares R, Alarcon JM, Kandel ER, Barco A (2007). cAMP response element-binding protein-mediated gene expression increases the intrinsic excitability of CA1 pyramidal neurons. J Neurosci..

[51] Midorikawa M, Sakaba T (2017). Kinetics of releasable synaptic vesicles and their plastic changes at hippocampal mossy fiber synapses. Neuron..

[52] Vaden JH, Banumurthy G, Gusarevich ES, Overstreet-Wadiche L, Wadiche JI (2019). The readily-releasable pool dynamically regulates multivesicular release. Elife..

[53] Oldani S, Moreno-Velasquez L, Faiss L, Stumpf A, Rosenmund C, Schmitz D, Rost BR (2021). SynaptoPAC, an optogenetic tool for induction of presynaptic plasticity. J Neurochem..

[54] Wozny C, Maier N, Fidzinski P, Breustedt J, Behr J, Schmitz D (2008). Differential cAMP signaling at hippocampal output synapses. J Neurosci..

[55] Santoro B, Chen S, Lüthi A, Pavlidis P, Shumyatsky GP, Tibbs GR, Siegelbaum SA (2000). Molecular and functional heterogeneity of hyperpolarization-activated pacemaker channels in the mouse CNS. J Neurosci..

[56] Lörincz A, Notomi T, Tamás G, Shigemoto R, Nusser Z (2002). Polarized and compartment-dependent distribution of HCN1 in pyramidal cell dendrites. Nat Neurosci..

[57] He C, Chen F, Li B, Hu Z (2014). Neurophysiology of HCN channels: from cellular functions to multiple regulations. Prog Neurobiol.

[58] Nolan MF, Malleret G, Dudman JT, Buhl DL, Santoro B, Gibbs E, et al. A behavioral role for dendritic integration: HCN1 channels constrain spatial memory and plasticity at inputs to distal dendrites of CA1 pyramidal neurons. Cell. 2004;119(5):719–32. 10.1016/j.cell.2004.11.020. 10.1016/j.cell.2004.11.020 15550252

[59] Boulton CL, McCrohan CR, O’Shaughnessy CT (1993). Cyclic AMP analogues increase excitability and enhance epileptiform activity in rat neocortex in vitro. Eur J Pharmacol.

[60] Sano M, Seto-Ohshima A, Mizutani A (1984). Forskolin supresses seizures induced by pentylenetrazol in mice. Experientia..

[61] Bender RA, Soleymani SV, Brewster AL, Nguyen ST, Beck H, Mathern GW, Baram TZ (2003). Enhanced expression of a specific hyperpolarization-activated cyclic nucleotide-gated cation channel (HCN) in surviving dentate gyrus granule cells of human and experimental epileptic hippocampus. J Neurosci..

[62] Lewis AS, Chetkovich DM. HCN channels in behavior and neurological disease: too hyper or not active enough? Mol Cell Neurosci. 2011;46(2):357–67. 10.1016/j.mcn.2010.11.007. 10.1016/j.mcn.2010.11.007 PMC3073601 21130878

[63] Noam Y, Bernard C, Baram TZ (2011). Towards an integrated view of HCN channel role in epilepsy. Curr Opin Neurobiol..

[64] Stegen M, Kirchheim F, Hanuschkin A, Staszewski O, Veh RW, Wolfart J (2012). Adaptive intrinsic plasticity in human dentate gyrus granule cells during temporal lobe epilepsy. Cereb Cortex..

[65] Yang S, Constantin OM, Sachidanandan D, Hofmann H, Kunz TC, Kozjak-Pavlovic V, Oertner TG, Nagel G, Kittel RJ, Gee CE, Gao S (2021). PACmn for improved optogenetic control of intracellular cAMP. BMC Biol..

[66] Stierl M, Penzkofer A, Kennis JTM, Hegemann P, Mathes T (2014). Key residues for the light regulation of the blue light-activated adenylyl cyclase from Beggiatoa sp. Biochemistry..

[67] Scheib U, Broser M, Constantin OM, Yang S, Gao S, Mukherjee S, et al. Rhodopsin-cyclases for photocontrol of cGMP/cAMP and 2.3 Å structure of the adenylyl cyclase domain. Nat Commun. 2018;9(1):2046. 10.1038/s41467-018-04428-w. 10.1038/s41467-018-04428-w PMC5967339 29799525

[68] Henß T, Nagpal J, Gao S, Scheib U, Pieragnolo A, Hirschhäuser A, et al. Optogenetic tools for manipulation of cyclic nucleotides functionally coupled to cyclic nucleotide-gated channels. Br J Pharmacol. 2021. 10.1111/bph.15445. 10.1111/bph.15445 33733470

[69] Feng G, Mellor RH, Bernstein M, Keller-Peck C, Nguyen QT, Wallace M, Nerbonne JM, Lichtman JW, Sanes JR (2000). Imaging neuronal subsets in transgenic mice expressing multiple spectral variants of GFP. Neuron..

[70] Heinrich C, Nitta N, Flubacher A, Müller M, Fahrner A, Kirsch M (2006). Reelin deficiency and displacement of mature neurons, but not neurogenesis, underlie the formation of granule cell dispersion in the epileptic hippocampus. J Neurosci..

[71] Haussler U, Bielefeld L, Froriep UP, Wolfart J, Haas CA (2012). Septotemporal position in the hippocampal formation determines epileptic and neurogenic activity in temporal lobe epilepsy. Cereb Cortex..

[72] Tulke S, Haas CA, Häussler U. Expression of brain-derived neurotrophic factor and structural plasticity in the dentate gyrus and CA2 region correlate with epileptiform activity. Epilepsia. 2019;60(6):1234–47. 10.1111/epi.15540. 10.1111/epi.15540 31121074

[73] Mahn M, Prigge M, Ron S, Levy R, Yizhar O (2016). Biophysical constraints of optogenetic inhibition at presynaptic terminals. Nat Neurosci..

[74] Esteller R, Echauz J, Tcheng T. Comparison of line length feature before and after brain electrical stimulation in epileptic patients. Conf Proc IEEE Eng Med Biol Soc. 2004:4710–3. 10.1109/IEMBS.2004.1404304. 10.1109/IEMBS.2004.1404304 17271360

[75] Heining K. Code for detecting and classifying epileptiform activity (EA). Zenodo. https://zenodo.org/record/4110614 (2020).

