# Supplementary material for: Long-term in vivo application of a potassium channel-based optogenetic silencer in the healthy and epileptic mouse hippocampus
Source: BMC Biol. 2022 Jan 14;20:18. doi: 10.1186/s12915-021-01210-1 (PMC8760681; doi:10.1186/s12915-021-01210-1)
Supplement: Supplementary file 1 — Additional file 1: Fig. S1. [Electrode and optic fiber positions in dorsal hippocampi of PACK mice]. Fig. S2. [Light application in mCherry mice does not alter neuronal activity in CA1]. Fig. S3. [Line length and gamma power decrease during reference recordings in bPAC mice]. Fig. S4- [The line length drops similarly in the first and second reference recording]. [file 12915_2021_1210_MOESM1_ESM.pdf]

Additional file 1

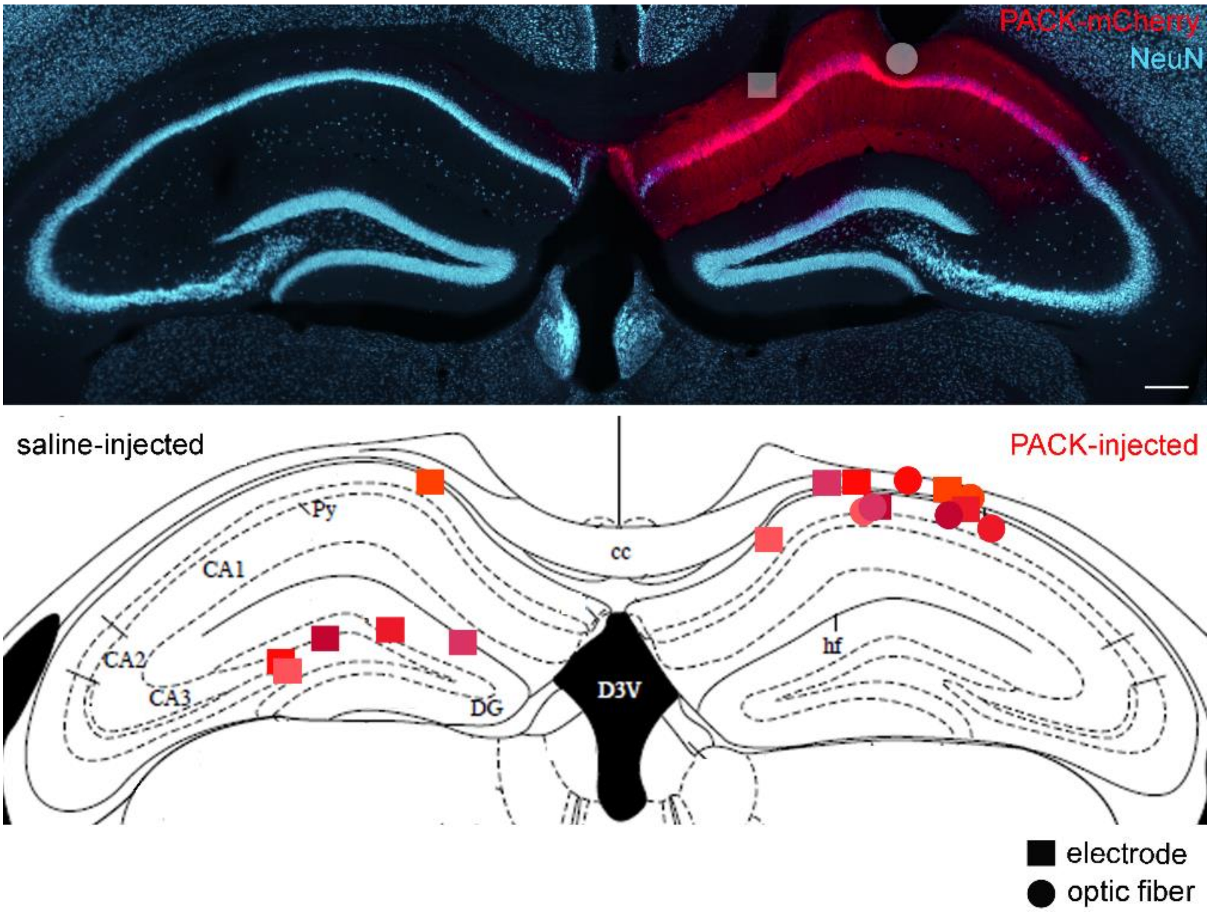

Figure S1. Electrode and optic fiber positions in dorsal hippocampi of PACK mice. (A) A representative NeuN-labeled hippocampal section, where the electrode (square) and optic fiber (circle) positions are visible in PACK-expressing right hippocampus. (B) Electrode and optic fiber positions in saline-injected and PACK-injected hippocampi of mice that were included in the LFP analysis (n=6, in different shades of red).

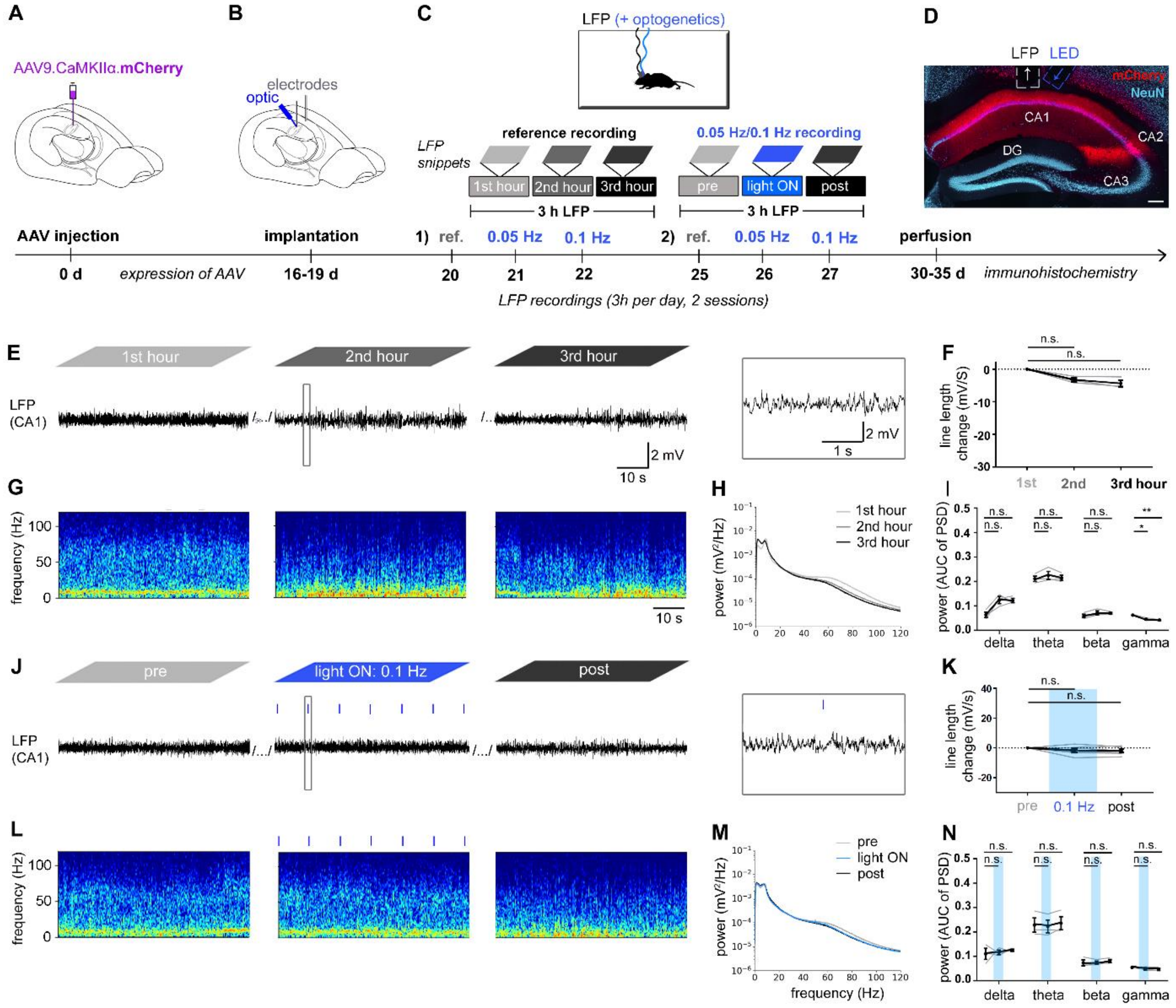

Figure S2. Light application in mCherry mice does not alter neuronal activity in CA1. Experimental design. We targeted mCherry to excitatory neurons in the CA1 area using the AAV9 vector. (B) Implantation, (C) recordings and (D) histological analysis were performed like in PACK mice. Scale bar 200 µm. (E) Representative LFP snippets from the first, second, and third hour of a reference recording. (F) The line length dropped slightly but not significantly in three hours. One-sample t-test (n=3, average of two recordings, grey lines). (G) The spectrograms, taken from the same time windows as LFP snippets. (H) Mean power spectral density (PSD) during the first (light grey), second (dark grey), and third (black) recording hour. (I) The power of delta (1-4 Hz), theta (4-12 Hz), and beta (12-30 Hz) oscillations did not change in three hours, whereas gamma (30-120 Hz) power dropped significantly. Two-way RM ANOVA, Dunnett's multiple comparison test. *p<0.05 , **p<0.01. (J) Representative LFP snippets of a 0.1 Hz recording show no response to the light application. (K) Line length was not changed by 0.1 Hz illumination. (L) The spectrograms were unaltered by light pulses. (M) The average PSD is nearly overlapping during pre-, light ON, and post-recordings, suggesting the light application did not affect the oscillations in the CA1 region. (N) The power of delta, theta, beta, and gamma oscillations remained the same across the three recording hours, not being altered by the illumination (shown in blue). Mean presented with SEM as error bars.

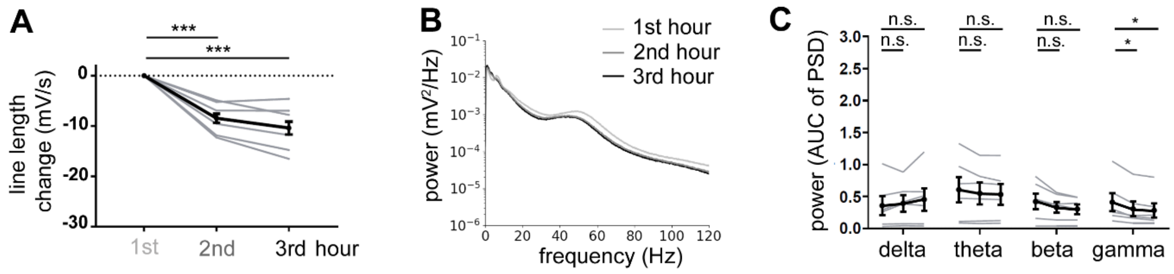

Figure S3. Line length and gamma power decrease during reference recordings in bPAC mice. (A) Line length drops significantly during the three-hour reference recordings. One-sample t-test (n=6 mice, average of two recordings, grey lines). (B) Mean PSD during the first (light grey), second (dark grey), and third (black) recording hour. (C) The power of delta, theta, and beta, oscillations did not change during the three hours, whereas the power of gamma oscillations declined significantly. Two-way RM ANOVA, Dunnett's multiple comparison test. *p<0.05, ***p<0.001. Mean presented with SEM as error bars.

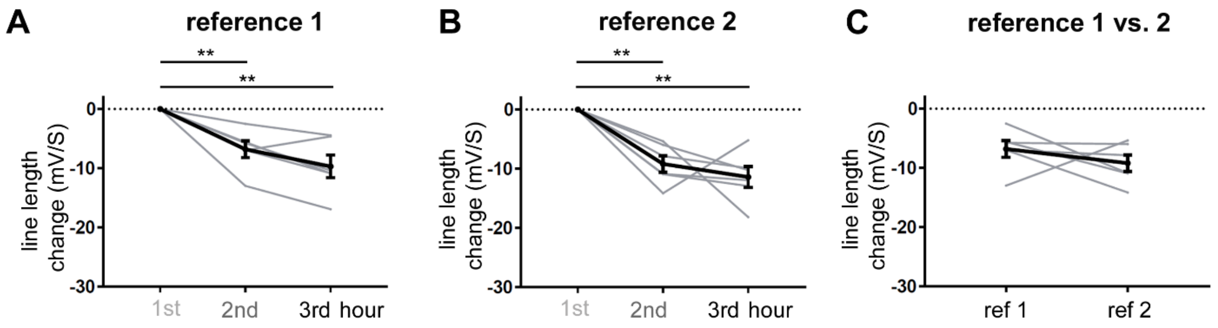

Figure S4. The line length drops similarly in the first and second reference recording. (A) Line length change in the first and (B) second reference recording (n=6 mice, average of two recordings, grey lines). One-sample t-test ** p<0.01. (C) The line length change from the first to the second hour is similar during the two reference recording sessions. Paired t-test. Mean presented in black with SEM as error bars.
