# Supplementary material for: Long-term in vivo application of a potassium channel-based optogenetic silencer in the healthy and epileptic mouse hippocampus
Source: BMC Biol. 2022 Jan 14;20:18. doi: 10.1186/s12915-021-01210-1 (PMC8760681; doi:10.1186/s12915-021-01210-1)
Supplement: Supplementary file 2 — Additional file 2: Table S1. [Results of the spectral analysis in saline PACK mice]. Table S2. [Results of the spectral analysis in saline bPAC mice]. [file 12915_2021_1210_MOESM2_ESM.pdf]

Additional file 2

Table S1. Results of the spectral analysis in saline PACK mice. We performed statistical analysis of the power in delta (1-4 Hz), theta (4-12 Hz), beta (12-30 Hz) and gamma (30-120 Hz) frequency bands. We performed multiple comparisons with two-way RM ANOVA and Dunnett's post hoc test to compare first recording hour with the second and third hour.

Session	Frequency band	Recording hour	Average power	Comparison with 1st h*
reference	delta	1	0.19 ± 0.04	-
		2	0.18 ± 0.03	n.s.
		3	0.17 ± 0.03	n.s.
	theta	1	0.51 ± 0.08	-
		2	0.39 ± 0.07	0.013
		3	0.38 ± 0.08	0.045
	beta	1	0.45 ± 0.05	-
		2	0.32 ± 0.04	0.0009
		3	0.29 ± 0.06	0.0003
	gamma	1	0.55 ± 0.07	-
		2	0.40 ± 0.06	0.0009
		3	0.36 ± 0.07	0.0005
0.1 Hz	delta	1	0.26 ± 0.06	-
		2	0.22 ± 0.08	n.s.
		3	0.21 ± 0.05	n.s.
	theta	1	0.65 ± 0.12	-
		2	0.43 ± 0.15	n.s.
		3	0.46 ± 0.10	0.025
	beta	1	0.74 ± 0.17	-
		2	0.29 ± 0.09	0.016
		3	0.40 ± 0.06	0.038
	gamma	1	0.72 ± 0.14	-
		2	0.26 ± 0.08	0.006
		3	0.45 ± 0.10	0.018

Table S2. Results of the spectral analysis in saline bPAC mice. We performed statistical analysis of the power in delta (1-4 Hz), theta (4-12 Hz), beta (12-30 Hz) and gamma (30-120 Hz) frequency bands. We performed multiple comparisons with two-way RM ANOVA and Dunnett's post hoc test to compare first recording hour with the second and third hour.

**Session**	**Frequency band**	**Recording hour**	**Average power**	**Comparison with 1st h***
reference	delta	1	0.36 ± 0.15	-
		2	0.39 ± 0.13	n.s.
		3	0.46 ± 0.17	n.s.
	theta	1	0.61 ± 0.20	-
		2	0.55 ± 0.17	n.s.
		3	0.54 ± 0.17	n.s.
	beta	1	0.42 ± 0.12	-
		2	0.33 ± 0.09	n.s.
		3	0.31 ± 0.08	n.s.
	gamma	1	0.41 ± 0.14	-
		2	0.31 ± 0.12	0.028
		3	0.29 ± 0.11	0.032
0.1 Hz	delta	1	0.34 ± 0.11	-
		2	0.40 ± 0.10	n.s.
		3	0.44 ± 0.15	n.s.
	theta	1	0.51 ± 0.15	-
		2	0.65 ± 0.24	n.s.
		3	0.46 ± 0.13	n.s.
	beta	1	0.26 ± 0.07	-
		2	0.77 ± 0.30	n.s.
		3	0.17 ± 0.05	n.s.
	gamma	1	0.39 ± 0.13	-
		2	0.73 ± 0.25	n.s.
		3	0.28 ± 0.10	0.036
